# Heat‐Dissipation Design and 3D Printing of Ternary Silver Chalcogenide‐Based Thermoelectric Legs for Enhancing Power Generation Performance

**DOI:** 10.1002/advs.202402934

**Published:** 2024-06-10

**Authors:** Keonkuk Kim, Seungjun Choo, Jungsoo Lee, Hyejin Ju, Soo‐ho Jung, Seungki Jo, So‐Hyeon Lee, Seongheon Baek, Ju‐Young Kim, Kyung Tae Kim, Han Gi Chae, Jae Sung Son

**Affiliations:** ^1^ Department of Chemical Engineering Pohang University of Science and Technology (POSTECH) Pohang 37673 Republic of Korea; ^2^ Department of Materials Science and Engineering Ulsan National Institute of Science and Technology (UNIST) Ulsan 44919 Republic of Korea; ^3^ Department of 3D Printing Materials Korea Institute of Materials Science (KIMS) Changwon 51508 Republic of Korea

**Keywords:** 3D printing, AgBiSe_2_, AgSbTe_2_, power generators, thermal designs, thermoelectric materials

## Abstract

Thermoelectric devices have received significant attention because of their potential for sustainable energy recovery. In these devices, a thermal design that optimizes heat transfer and dissipation is crucial for maximizing the power output. Heat dissipation generally requires external active or passive cooling devices, which often suffer from inevitable heat loss and heavy systems. Herein, the design of heat‐sink integrated thermoelectric legs is proposed to enhance heat dissipation without external cooling devices, realized by finite element model simulation and 3D printing of ternary silver chalcogenide‐based thermoelectric materials. Owing to the self‐induced surface charges of the synthesized AgBiSe_2_ (n‐type) and AgSbTe_2_ (p‐type) particles, these particle‐based colloidal inks exhibited high viscoelasticity, which enables the creation of complex heat‐dissipation architectures via 3D printing. Power generators made from 3D‐printed heat‐dissipating legs exhibit higher temperature differences and output power than traditional cuboids, offering a new strategy for enhancing thermoelectric power generation.

## Graphical abstract

The computational design and 3D printing of heat‐sink‐integrated self‐heat‐dissipation thermoelectric legs for enhancing power‐generating performance are reported. The viscoelastic inks of n‐type AgBiSe_2_ and p‐type AgSbTe_2_ are formulated and 3D‐printed to create the designed architectures with the maximum *ZT* values of 0.5 and 1.2. The fabricated power generator exhibits higher output power than that of a cuboid‐based device by 80%.

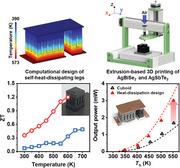

## 1 Introduction

Thermoelectric (TE) devices have attracted tremendous attention because of their potential use as sustainable energy harvesters. These devices can directly convert waste heat from diverse heat sources, in the housing and industrial sectors, automobiles, and ships, into electricity.^[^ ^1^ ^]^ In TE power generation systems, the thermal design is essential for optimizing effective heat transfer and dissipation, thereby maximizing the power output or efficiency.^[^ ^2^ ^]^ To date, the design process for practical operation has mainly been conducted by designing external heat‐dissipation devices rather than using the TE device itself for heat dissipation. For example, a heat sink is a passive heat‐exchange cooler typically chipped on the cold side of a TE device for heat dissipation. This device has been researched to be designed with various design parameters such as pin spacing, diameter, height, and orientation for efficient heat dissipation under convective conditions.^[^ ^3^ ^]^ However, combining TE devices with external cooling components often leads to inevitable thermal loss at the TE‐heat sink interface and renders the entire system very heavy. Moreover, these challenges become more pronounced when attempting to customize TE devices for heat sources with irregular shapes, which limits the versatility of TE devices for a wide range of heat sources. A possible solution to these challenges is to find a means of maximizing heat dissipation within the TE device itself, eliminating the need for an external cooling component.

TE devices comprise p‐ and n‐type semiconductors joined together in a thermocouple configuration. Conventionally, the manufacturing process for these devices involves multiple steps, including synthesis of p‐ and n‐type materials, dicing, metallization, and assembly into substrates. This conventional procedure presents certain challenges, particularly in terms of the limited shape, because it is technically impossible to produce any complex shape other than a cube using the top‐down dicing process in the context of mass production. Recently, 3D printing has emerged as a cost‐effective method for manufacturing bulk‐scale TE materials and devices.^[^ ^4^ ^]^ This process can provide flexibility in the shape and dimensional control of TE materials and facilitate the production of TE materials and devices with customized designs. Recent studies reported a direct ink 3D printing process for diverse TE materials with complex designs such as tubes, honeycombs, and lattices.^[^ ^5^ ^]^ Despite recent advances, this technology is still in the development phase, and further advancements combined with 3D thermal design are required to adapt it to diverse thermal environments. This is in stark contrast to the well‐established field of 3D printing in mechanics, where a wide range of design tools has been extensively studied and applied to specific applications over many years.

Ternary silver chalcogenides are regarded as efficient mid‐temperature‐operable TE materials and are potential alternatives to lead‐chalcogenide‐based materials.^[^ ^6^ ^]^ These crystals have intrinsically ultralow thermal conductivities because of their extreme lattice anharmonicity and cation disorder (Figure S1, Supporting Information),^[^ ^7^ ^]^ and materials with specific compositions, such as n‐type AgBiSe_2_ and p‐type AgSbTe_2_ have emerged as promising TE materials with high *ZT* values.^[^ ^8^ ^]^ Here, we explore the heat‐sink‐inspired heat‐dissipation design and 3D printing of ternary silver‐chalcogenide‐based TE materials and power generators. We designed a topology for TE legs with multiple pins and optimized the design parameters of the number, diameter, and spacing of pins to maximize heat‐dissipation efficiency. To fabricate the designed architecture, we developed an extrusion‐based 3D printing process for the TE materials. The controlled compositions of the AgBiSe_2_ and AgSbTe_2_ compounds induced intrinsic surface charges on the TE particles without any additives, enabling the direct 3D writing of these inks to print 3D architectures. These impurity‐free inks allow efficient sintering of the 3D‐printed TE materials, with enhanced *ZT* values of up to 0.49 for the n‐type AgBiSe_2_ at 700 K and 1.20 for the p‐type AgSbTe_2_ at 600 K. The 3D‐printed heat‐dissipating TE legs were chipped in power‐generating modules and comparatively evaluated with typical cuboid TE legs, significantly enhancing the temperature differences across a designed TE leg and eventually the output powers. These results demonstrate the feasibility of the proposed design toward the enhancement of power performance of TE generators (TEGs).

## 2 Results and Discussion

### 2.1 Heat‐Dissipation Design of TE Materials

**Figure 1:**
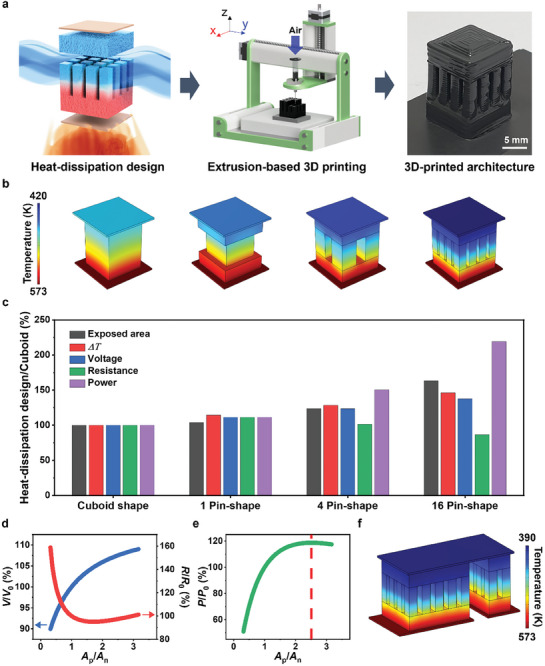
Heat‐dissipation design of TE leg. a) Schematic showing the overall processing for the fabrication of the heat‐dissipation‐designed TE leg. b) The designs and the simulated temperature distributions of cuboid, 1‐, 4‐, and 16‐pin‐shaped AgBiSe_2_ TE legs. c) The percentage of exposed surface area, temperature difference, output voltage, electrical resistance, and output power of the heat‐dissipation‐designed shapes compared to those of the cuboid AgBiSe_2_ TE leg. The percentage of the d) output voltage and electrical resistance and e) output power compared to that of the identical ratio as a function of *A*_p_/*A*_n_. f) The optimum TEG design constructed with a twenty‐pin p‐type TE leg and an eight‐pin n‐type TE leg and its simulated temperature distributions.

To design the topology of the heat‐sink‐inspired TE legs for optimum heat dissipation, we developed a 3D finite element method (FEM) model for comparative calculations of the power‐generating performances of the designed TE legs with those of typical cuboid TE legs. The maximum output power (*P*_max_) is expressed as

(1) $$P_{\max}=\frac{{\left(\alpha_{p}-\alpha_{n}\right)}^{2}\Delta T^{2}}{4R}$$

where α_*i*_ is the Seebeck coefficient of the leg, *i* = n, p, and *R* is the internal electrical resistance of the TEG. Heat‐dissipative designs increase Δ*T* through reduction of the temperature on the cold side of the TE, thereby increasing *P*_max_.^[^ ^9^ ^]^ From a heat‐dissipation perspective, a heat sink shape with multiple pins have a larger exposed surface area, which in turn increases the ability to dissipate thermal energy by convection from each pin to the surrounding medium. The dissipated heat (*Q*) is defined by the following equation:

(2) $$Q=hA\left(T_{\mathrm{surf}}-T_{\mathrm{amb}}\right)$$

where *h* is the convection coefficient, *A* is the exposed surface area, *T*_surf_ is the pin surface temperature, and *T*_amb_ is the ambient‐medium temperature.^[^ ^10^ ^]^ In this study, we propose a heat‐dissipation design for TE legs, inspired by the heat sink shape, to create higher temperature differences and therefore higher output powers (**Figure** **1**a). The TE materials used in this design model and in the 3D printing fabrication were AgBiSe_2_ and AgSbTe_2_ compounds for the n‐ and p‐type semiconductors, respectively. Details of the material properties used in the simulations will be discussed in the following sections. The boundary conditions were set with the hot‐side temperature fixed at 573 K, considering the material properties, whereas the other surfaces were cooled by natural convection with a convection coefficient of 5 W m^−2^ K^−1^. In our FEM models, we comparatively investigated the effect of the pin numbers in TE legs (cuboid, one pin, four pins, and sixteen pins) on the exposed surface area, distribution of temperature, electrical resistance, output voltage, and power (Figure 1b,c and Figures S2–S4, Supporting Information). As manifested in Figure 1c, the Δ*T* and output voltages of the TE legs enhanced with an increasing number of pins, clearly indicating the heat‐dissipation effect at the pins.^[^ ^2b^ ^]^ For the sixteen‐pin model, the Δ*T* and output voltages increased by 46% and 37%, compared with those of the cuboid TE leg with an identical height and volume. The electrical resistances fluctuate slightly with the number of pins because the electrical conductivity of the TE material is strongly dependent on the temperature, and the created temperature ranges across the models differ from one another. Consequently, owing to the increased Δ*T* and output voltage, the sixteen‐pin model showed a 119% increase in output power than the cuboid model.

In addition to the heat‐dissipation design, we further optimized the cross–sectional area ratio of the p‐ and n‐type TE legs (*A*_p_/*A*_n_) to minimize the mismatch between the electrical and thermal resistances (Figure S5, Supporting Information). In this computation, we assumed that the optimum cross–sectional area ratio was similar for both the cuboid and heat‐dissipation designs.^[^ ^5d^ ^]^ In addition, we set constant total volumes for the n‐ and p‐type legs. The FEM simulation shows that an increase in the *A*_p_/*A*_n_ ratio leads to an increase in the thermal resistance of the TEG owing to the lower thermal conductivity of the p‐type material compared to that of the n‐type material, resulting in a higher temperature difference and consequently higher output voltage (Figure 1d). However, the electrical resistance is minimal when *A*_p_/*A*_n_ is close to 1.7, at which point the mismatch in the electrical resistances of the n‐ and p‐type legs becomes minimal. Consequently, a pair of cuboid TE legs was expected to produce the maximum *P* when *A*_p_/*A*_n_ was ≈2.5 (Figure 1e). According to the simulation results of heat‐dissipation designs and cross–sectional area ratios, we designed an optimum device that was constructed with a twenty‐pin p‐type leg and an eight‐pin n‐type leg to be 3D‐printed (Figure 1f). The final design is predicted to exhibit an output power 102% higher than that of the cuboidal TEG with an identical cross–sectional ratio and 16% higher than that of the heat‐dissipation‐designed TEG with an unoptimized cross–sectional ratio (Figure S6, Supporting Information).

### 2.2 Formulation of Viscoelastic Colloid Inks of Ternary Ag Chalcogenides

**Figure 2:**
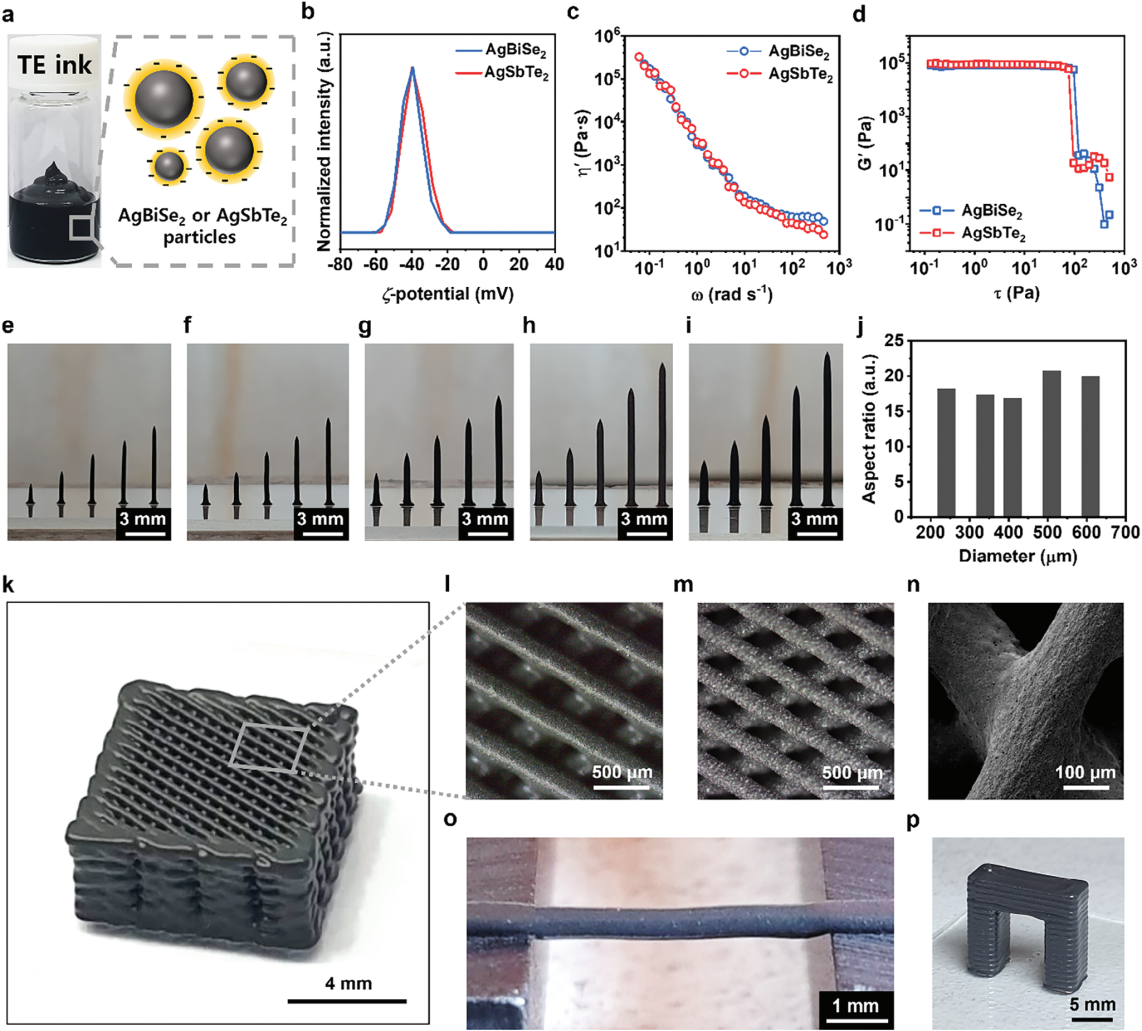
Viscoelasticity and 3D printing of ternary Ag chalcogenide TE inks. a) Photograph and illustration showing the synthesized ternary Ag chalcogenide TE ink for extrusion‐based 3D printing. b) *ζ*‐potential of the AgBiSe_2_ and AgSbTe_2_ TE inks. c) Dynamic viscosity (*η*′) obtained from frequency sweep test and d) storage modulus (*G*′) obtained from stress sweep test of the AgBiSe_2_ and AgSbTe_2_ TE inks. Photographs showing the 3D‐printed standing TE filaments with diameters of e) 240, f) 340, g) 410, h) 510, and i) 610 µm. j) The maximum aspect ratio of the 3D‐printed standing TE filaments at each diameter. k) Photograph showing the 3D‐printed lattice structure. OM images of l) 3D‐printed and m) sintered filaments forming the lattice structure. n) High‐magnification SEM image of the lattice structure. o) Photograph showing the 3D‐printed parallel filament without sagging. p) Photograph showing the 3D‐printed bridge structure.

To realize the heat‐dissipation‐designed TE leg by 3D printing, we developed the extrusion‐based 3D printing process using colloidal inks based on AgBiSe_2_ and AgSbTe_2_ particles dispersed in a glycerol medium (**Figure** **2**a), which maintained colloidal stability for more than 10 days (Figure S7, Supporting Information). The glycerol has a high viscosity, which ensures the effective dispersibility of TE particles.^[^ ^5^ ^]^ Furthermore, it functions as a humectant in the TE inks, facilitating the cohesion of the individual printed layers into a unified structure.^[^ ^11^ ^]^ The particles were synthesized by mechanical alloying, and their X‐ray diffraction (XRD) patterns corresponded to those of bulk AgBiSe_2_ and AgSbTe_2_ references without any appreciable peaks related to impurities (Figure S8, Supporting Information). These inks should have appropriate viscoelastic and rheological properties to ensure 3D printability, in which the ink flows upon deposition while maintaining its structural integrity after deposition.^[^ ^12^ ^]^ Such viscoelastic properties of inks are usually obtained by the addition of organic rheological modifiers; however, this can degrade the functional properties of 3D‐printed products, especially the electrical properties of TE materials, owing to the insulating nature of organic binders.^[^ ^13^ ^]^ Our group reported 3D‐printable viscoelastic inks of TE particle colloids without the use of organic rheological modifiers, for example, all‐inorganic colloid particles tailored by surface‐stabilizing anions such as Sb_2_Te_4_^2−^ chalcogenidometallate anions and Se_8_^2−^ polyanions, or electronic doping, which induce strong surface charges.^[^ ^5^ ^]^ Such surface charges significantly improve the viscoelasticity of the particle colloids owing to the electroviscous effect. The local electric field induced at the particle surfaces can weaken their mobility in the liquid medium, eventually enhancing both the viscosity and elasticity of the colloids. In this study, we found that the stoichiometric AgBiSe_2_ and AgSbTe_2_ particles intrinsically have strong surface charges, as manifested in the zeta(*ζ*)‐potential spectrum, in which −39.9 mV for AgBiSe_2_ and −39.4 mV for AgSbTe_2_ were observed (Figure 2b). These intrinsic surface charges facilitate the formation of purely inorganic particle colloids and do not require additives that potentially degrade their electrical and thermal properties. The dynamic light scattering (DLS) spectrums of the particles reveal that both TE particles have sizes ranging from ≈200 to 800 nm (Figure S9, Supporting Information), which can ensure colloidal dispersion.

To understand the intrinsic surface charges of AgBiSe_2_ and AgSbTe_2_ colloid particles, we conducted both the *ζ*‐potential and X‐ray photoelectron spectroscopy (XPS) analysis on particles (Figures S10–S12, Supporting Information) by varying the chalcogen contents in the synthesized AgBiSe_2+_ *_x_* and AgSbTe_2+_ *_x_* particles (*x* = −0.1, 0, and 0.1). The *ζ*‐potential spectrum of AgBiSe_2+x_ inks reveals that the increase in the Se content led to an increase in the absolute *ζ*‐potential values. In the XPS spectrum of the AgBiSe_2+x_ particles, the peaks in the Ag 3d and Se 3d regions did not substantially change with a change in Se content. However, in the Bi 4f region, with increasing the Se content, the metallic Bi peaks in the Bi 4f region are pronounced, while Bi_2_O_3_ peak intensity significantly decreases. Since the exposed atoms at the particle surfaces are more easily oxidized, these results suggest that an increase in the Se content exposes a greater number of Se atoms, whereas a smaller number of Bi atoms are exposed at the particle surfaces, which could induce stronger negative surface charges in the AgBiSe_2+_ *_x_* particles. In contrast, for AgSbTe_2+_ *_x_* particles, the *ζ*‐potential spectrum shows that the surface charges are not changed regardless of variations in the Te content. Moreover, the XPS spectrum revealed that the oxidation of Te intensified with an increase in the Te content, whereas we did not observe substantial changes in the peaks for Ag and Sb. This analysis indicates that the surfaces of AgSbTe_2_ are preferentially terminated by Te ions rather than by Ag or Sb, resulting in consistent negative surface charges independent of the Te content.

Such strong surface charges in TE‐particle‐based colloidal inks are directly reflected in their excellent rheological viscoelasticity. The rheological properties of the inks were characterized by measuring their angular‐frequency‐dependent dynamic viscosity, shear‐stress‐dependent storage modulus, and thixotropic properties using a three‐interval test. These tests provide a comprehensive evaluation of the rheological properties of the ink and their implications for printability. The steady‐state dynamic viscosities reach 3.38 × 10^5^ Pa·s for AgBiSe_2_ and 3.23 × 10^5^ Pa·s for AgSbTe_2_ inks at the angular frequency of 0.06 rad s^−1^ (Figure 2c), which are comparable or even higher values than the reported values for 3D direct writable Bi_2_Te_3_‐based inks.^[^ ^5e^ ^]^ The storage moduli of inks exhibit 7.0–8.1 × 10^5^ Pa for AgBiSe_2_ and 8.2–9.6 × 10^5^ Pa for AgSbTe_2_ in the wide range of shear stress (Figure 2d). Interestingly, the critical stress values of these inks were significant, 96.54 Pa for AgBiSe_2_ and 76.32 Pa for AgSbTe_2_. These results indicate that the inks can withstand high shear forces before undergoing structural deformation. The ability to tolerate high shear stresses is essential for maintaining the structural integrity of printed objects, thereby enabling high‐resolution printing to create complex geometries. During thixotropic tests (Figure S13, Supporting Information), the ink displayed rapid recovery after deformation within the shear stress range in which yielding occurred. This implies that the ink quickly regained its original viscosity after being subjected to shear stress, contributing to printability by ensuring a stable ink flow and consistent layer deposition.^[^ ^12^ ^]^

### 2.3 3D Printing of Ternary Ag Chalcogenide TE Materials

The extremely high viscoelasticity of our inks allowed us to create various 3D architectures by directly writing TE filaments according to a designed computer‐aided model (CAD). These filaments, created in a single pass, exhibited structural retention and smooth surfaces (Figure S14, Supporting Information). Moreover, the diameters of the as‐printed filaments were precisely controlled by various printing parameters such as printing speed and dispensing pressure, in which faster printing and lower pressure led to thinner filaments (Figure S15, Supporting Information). This demonstrates the applicability of the current process at multiple scales ranging from hundreds of micrometers to millimeters. The photographs show uniformly written filaments with different diameters and aspect ratios (Figure 2e–i), with the maximum aspect ratio reaching 15–20 at each diameter (Figure 2j). Based on the printability of single microfilaments, the lattice structures of AgBiSe_2_ and AgSbTe_2_ as examples of 3D architectures, were printed using layer‐by‐layer deposition. Photographs and optical microscope (OM) images (Figure 2k–m) show that the 3D lattice is constructed by uniformly deposited filaments with a diameter of ≈210 µm. These structures exhibited excellent structural retention without merging at the junction or collapsing, indicating the capability of the ink to maintain the integrity of the printed structure. Upon heating for sintering of the TE materials, the 3D structures exhibited homogeneous sintering shrinkage, e.g., the diameter of filaments uniformly contracted from 210 to 165 µm. As seen in the scanning electron microscopy (SEM) image (Figure 2n), the filaments at the junction were well fused without crack formation or structural distortion after heat treatment.

Another important function of 3D ink writing is bridging with a single TE filament, which is crucial for realizing a heat‐dissipation design by 3D printing. When a single filament is printed over an empty space, it sustains a gravitational force that pulls the filament downward, i.e., it sags or droops in the unsupported span between two connected regions.^[^ ^14^ ^]^ Accordingly, we investigated the bridging printing of our inks between two graphite blocks by varying the block‐to‐block distance from 3 to 7 mm (Figure S16, Supporting Information). The printing nozzle moved from the center of one block to the other during ink extrusion. The bridging TE filament was well sustained up to a distance of 6 mm (Figure 2o), however, the ink started to sag when the distance was longer than 7 mm. Based on this bridging capability, we further 3D‐printed the bridge architecture constructed using two legs with a high aspect ratio and one long bridge (Figure 2p). These results demonstrate the excellent 3D printability of our inks, which fulfill all the required functions of 3D printing to build heat‐dissipation‐designed TE legs in terms of printing resolution and structural retention of the printed structure. The entire 3D printing fabrication process of the heat‐dissipation‐designed TE leg is shown in Video S1 (Supporting Information).

The 3D‐printed materials were further heat‐treated to ensure good TE performance and mechanical robustness. The heat treatment conditions were optimized to maximize the relative densities of both materials by sintering the particles. Here, the 3D‐printed AgBiSe_2_ and AgSbTe_2_ samples were denoted by ABS# and AST# (# is the sintering duration time in hours). The sintering temperature window for AgBiSe_2_ was found to be quite narrow, from 973 to 1003 K. Below 973 K, the 3D‐printed sample was not effectively sintered, whereas the melting temperature of the AgBiSe_2_ crystal was 1038 K. Accordingly, the sintering temperature was optimized at 988 K, and we investigated the effect of the sintering duration. As the sintering duration was extended from 1 to 5 h, the relative density of the AgBiSe_2_ samples gradually increased from ≈65% to 88% and became saturated (Figure S17a, Supporting Information). The XRD patterns of the ABS1, ABS3, and ABS5 show a hexagonal AgBiSe_2_ pattern with peaks corresponding to the secondary phases of Ag_2_Se and Bi_4_Se_3_ (Figure S18a,c, Supporting Information). Since the as‐ball‐milled AgBiSe_2_ particles have the cubic phase, which is known to be unstable at room temperature,^[^ ^7^, ^15^ ^]^ the cubic phase was transformed to the stable hexagonal phase during the 3D printing and sintering processes. It is speculated that the formation of unstable phases results from the harsh synthetic condition of the mechanical alloying. The peaks indexed to the Ag_2_Se and Bi_4_Se_3_ phases were more pronounced in the XRD patterns as the sintering time increased. This can be attributed to Se evaporation, which induces the formation of Se‐deficient phases of Ag_2_Se and Bi_4_Se_3_ inside the AgBiSe_2_ matrix at equilibrium.^[^ ^16^ ^]^ The optimal sintering temperature for AgSbTe_2_ is 803 K, below which the sample is not sintered and exhibits a powder‐like state. However, unlike AgBiSe_2_, the relative density of AgSbTe_2_ was less affected by the sintering duration and rapidly saturated to ≈68% after 1 h of sintering (Figure S17b, Supporting Information). The XRD patterns of the AST1, AST5, and AST 9 correspond well with those of the cubic AgSbTe_2_ reference (Figure S18b, Supporting Information), which are the most stable phases at room temperature. Unlike the AgBiSe_2_ particle, the phase‐transition of AgSbTe_2_ was not observed during the 3D printing and sintering processes. Tiny peaks corresponding to the secondary phase of Ag_2_Te were observed in the XRD patterns of all the samples, regardless of the sintering conditions (Figure S18d, Supporting Information). The SEM images show that the printed and dried samples of both AgBiSe_2_ and AgSbTe_2_ were powder‐like, whereas the heat‐treated samples exhibited well‐fused and densified grains, indicating effective sintering (Figure S19, Supporting Information). The pore size and size distribution in the sintered samples were further analyzed by measuring the sizes of more than 200 pores in the SEM images. According to the statistical analysis of the pore size (Figure S20, Supporting Information), the average pore size of the AgBiSe_2_ samples decreased from 5.56 µm in the ABS1 to 2.49 µm in the ABS5, indicating the densification of TE particles during sintering and consistent with the observed reduction in porosity. Conversely, the sintering duration on the average pore size of the AgSbTe_2_ samples was not so significant, with a slight decrease from 3.39 µm in the AST1 to 2.66 µm in the AST9. This trend corresponds with the densification behavior of the AgSbTe_2_ samples, where the density remained relatively unchanged with extended sintering duration. The entire printed architecture underwent volume shrinkage of ≈25% homogeneously in all directions, thus allowing us to achieve the final 3D architecture according to the CAD‐based predesign.

### 2.4 TE and Mechanical Properties of 3D‐Printed Ternary Ag Chalcogenide Materials

**Figure 3:**
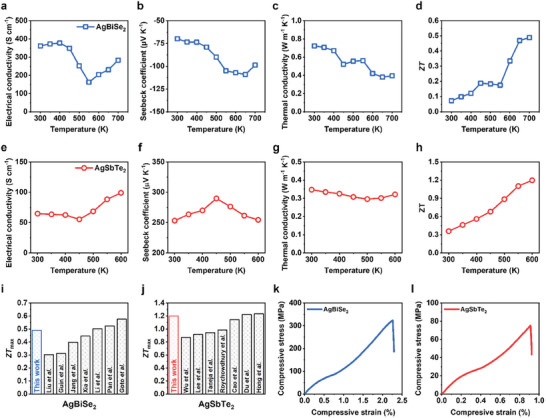
TE and mechanical properties of 3D‐printed ternary Ag chalcogenide TE samples. Temperature‐dependent a) electrical conductivity, b) Seebeck coefficient, c) thermal conductivity, and d) *ZT* of the 3D‐printed AgBiSe_2_ sample. Temperature‐dependent e) electrical conductivity, f) Seebeck coefficient, g) thermal conductivity, and h) *ZT* of the 3D‐printed AgSbTe_2_ sample. Comparison of the maximum *ZT* values between this work and the reported pristine i) AgBiSe_2_ and j) AgSbTe_2_ samples. Compressive stress–strain curve of the 3D‐printed k) AgBiSe_2_ and l) AgSbTe_2_ samples.

We measured the temperature‐dependent electrical and thermal properties of 3D‐printed and sintered AgBiSe_2_ and AgSbTe_2_ at temperatures ranging from 300 to 600–700 K (**Figure** **3**a–h). The AgBiSe_2_ sample exhibited the 36200 S m^−1^ of the electrical conductivity and −69.75 µV K^−1^ of the Seebeck coefficient at room temperature. Fluctuations in the electrical properties at 400–600 K were observed at elevated temperatures. This phenomenon is attributed to the intrinsic phase transitions of the AgBiSe_2_ compound, where the first transition from hexagonal to rhombohedral occurred at 460 K, and the second transition from hexagonal to cubic occurred at 580 K.^[^ ^8b^ ^]^ Such phase transitions generally cause abrupt changes in the carrier concentration, resulting in fluctuations in electrical properties. In our samples, the electrical conductivity increased slowly from room temperature to 460 K and rapidly decreased in the temperature range of 460 to 580 K, in agreement with the reported properties of AgBiSe_2_ compounds, because rhombohedral AgBiSe_2_ behaves like a metallic phase. Above 580 K, a phase transition to the cubic phase occurs, and the temperature dependence of the electrical conductivity becomes positive. The temperature‐dependent Seebeck coefficients of the AgBiSe_2_ samples also showed a similar fluctuation in their electrical conductivities owing to phase transitions.

We further investigated the influence of the sintering duration on the TE properties, because sintering involves Se evaporation and resulting compositional changes. In particular, Se vacancies are known to act as electron donors in AgBiSe_2_,^[^ ^17^ ^]^ which allows the control of the electron concentration to optimize the thermoelectric properties. Moreover, the presence of the Bi_4_Se_3_ secondary phase, which has higher mobility than the AgBiSe_2_ matrix, causes enhanced electron transport properties, resulting in an overall improvement in mobility.^[^ ^8g^ ^]^ To verify this effect, we conducted Hall effect measurements, by which the measured electron concentration increased from 0.31 × 10^19^ to 3.11 × 10^19^ cm^−3^ with an increase in sintering time from 1 to 5 h (Figure S21, Supporting Information). Meantime, the electron mobility also increased from 2.05 to 72.75 cm^2^ V^−1^ s^−1^. The increase in carrier mobility can be attributed to densification and the formation of the Bi_4_Se_3_ secondary phase under longer sintering conditions, as seen in the SEM images showing less porous microstructures in the samples sintered for a longer duration (Figure S22, Supporting Information). Accordingly, with a shorter sintering duration, the electrical conductivity decreased, whereas the Seebeck coefficient increased, owing to the lower electron concentration (Figure S23a,b, Supporting Information). Overall, the power factor of the ABS1 and ABS3 sintered at a shorter time was inferior to the ABS5. Therefore, we chose the sintering condition of 5 h at 988 K, under which the fabricated sample exhibited the maximum power factor of 2.76 µW cm^−1^ K^−2^ at 700 K (Figure S23e, Supporting Information).

The temperature‐dependent thermal conductivity of the AgBiSe_2_ sample generally decreased with increasing temperature over the entire measurement temperature range and reached its lowest value in the temperature region above 600 K.^[^ ^8b^ ^]^ The minimum thermal conductivity was found to be 0.38 W m^−1^ K^−1^ at 650 K. The samples sintered for shorter durations exhibited similar temperature‐dependent behaviors, but lower values than the ABS5, owing to the lower electronic thermal conductivity (Figure S23c, Supporting Information). Moreover, similar temperature‐dependent fluctuations in the electrical properties were observed in all samples. We calculated the lattice thermal conductivity (*κ*_L_) by subtracting the electronic thermal conductivity (*κ*_e_) from the total thermal conductivity. *κ*_e_ relies on the Wiedermann‐Franz law (*κ*_e_ = *LσT*), where *L* is the Lorenz number. *κ*_L_ shows similar trends to the total thermal conductivity, and a minimum value of 0.11 W m^−1^ K^−1^ was observed in the ABS5 (Figure S23g, Supporting Information). This is mainly due to effective phonon scattering by the fully disordered distribution of Ag^+^ and Bi^3+^ ions in the cubic phase of AgBiSe_2_ and the interfaces at the secondary phases of Ag_2_Se and Bi_4_Se_3_.^[^ ^18^ ^]^ We further calculated the *κ*_L_ of our 100% dense samples from the effective medium theory. The calculated *κ*_L_ ranged from 0.27 to 0.7 W m^−1^ K^−1^ in the ABS1 (Figure S23h, Supporting Information), which is in a range similar to reported values.^[^ ^8^, ^19^ ^]^

Unlike the AgBiSe_2_ samples, which showed a clear distinction of the electrical and thermal properties according to the sintering duration, the AgSbTe_2_ samples sintered at 803 K exhibited similar properties at all measurement temperatures. This phenomenon indicates that the microstructures of the sintered AgSbTe_2_ were almost identical in all samples, regardless of the sintering duration, as seen in the SEM images (Figure S24, Supporting Information). This was further supported by the identical densities of all samples and the existence of the Ag_2_Te secondary phase. Also, the compositional change associated with Te evaporation was not observed in the AgSbTe_2_ samples, owing to the relatively low vapor pressure of Te. In all the samples, fluctuations in both the electrical conductivity and Seebeck coefficient were observed at 420 K (Figure S25a,b, Supporting Information) owing to the monoclinic‐to‐cubic phase transition of the Ag_2_Te precipitate.^[^ ^8^, ^20^ ^]^ Accordingly, we chose the AST1 for the fabrication of the TE devices, considering processing time and productivity. The electrical conductivity and the Seebeck coefficient of the AST1 were 6500 S m^−1^ and 253 µV K^−1^ at room temperature, respectively. The power factor reached 6.42 µW cm^−1^ K^−2^ at 600 K (Figure S25e, Supporting Information). The peak was also found in the thermal conductivity, with a minimum thermal conductivity of 0.29 W m^−1^ K^−1^ at 500 K (Figure S25c, Supporting Information). The lattice thermal conductivity was calculated by the method previously mentioned, and the minimum *κ*_L_ value of 0.23 m^−1^ K^−1^ was observed in the AST1 (Figure S25g, Supporting Information). Such a low thermal conductivity can also be attributed to the disordered distribution of Ag and Sb cations, as in the case of AgBiSe_2_. Further calculation for obtaining the *κ*_L_ of the 100% dense sample gives the calculated *κ*_L_ values ranging from 0.4 to 0.6 W m^−1^ K^−1^ (Figure S25h, Supporting Information), similar to previously reported values.^[^ ^8d,j^ ^]^

The *ZT* values were calculated from the measured electrical and thermal properties. In both materials, the *ZT* value generally increased with increasing temperatures and reached the maximum values of 0.49 for n‐type AgBiSe_2_ at 700 K and 1.20 for p‐type AgSbTe_2_ at 600 K. These maxima are comparable to or slightly higher than the reported values for undoped AgBiSe_2_ and AgSbTe_2_ bulk samples synthesized by conventional methods, such as solid‐state reaction, hot pressing, and spark plasma sintering (Figure 3i,j).^[^ ^8^, ^17^, ^21^ ^]^

The 3D‐printed samples were further subjected to uniaxial compression tests at room temperature. In both the AgBiSe_2_ and AgSbTe_2_ samples, the compressive stress increased with increasing strain and then suddenly decreased, indicating a direct brittle fracture (Figure 3k,l). The compressive strengths of the 3D‐printed AgBiSe_2_ and AgSbTe_2_ are 324.0 and 75.25 MPa, respectively. These values are similar or superior to those of other conventional bulk TE materials, such as Bi_2_Te_3_, Cu_2_Se, SnSe, and PbTe (Figure S26, Supporting Information).^[^ ^22^ ^]^ Young's moduli of the samples were calculated from the ratio of the change in stress and the strain (Δ*σ*/Δ*ε*) at the failure point due to their brittle features. The calculated Young's moduli of 3D‐printed AgBiSe_2_ and AgSbTe_2_ are 14.34 and 8.258 GPa, respectively. These values were lower than those of conventional TE materials.^[^ ^23^ ^]^ The high compressive strengths and low Young's moduli indicate high toughness, which is the ability to absorb mechanical energy up to the point of failure; therefore, it is helpful in improving durability and operational reliability in practical thermoelectric applications. We further compare the mechanical properties of the samples printed along the in‐plane and cross‐plane orientations because the part is printed layer‐by‐layer, and the layer‐to‐layer adhesion sometimes is not as strong as that in‐plane. As shown in the stress–strain curves of AgBiSe_2_ (Figure S27e, Supporting Information), each sample exhibited nearly identical compressive strain and stress regardless of the printing orientation, indicating the isotropic mechanical properties. This suggests that the interlayer bonding is strong due to the well‐merged filaments, which is consistent with the high measured relative density of AgBiSe_2_. Meanwhile, the stress–strain curves of AgSbTe_2_ show that slight anisotropic properties depending on their printing orientations (Figure S27f, Supporting Information). The compressive strength of the sample printed in the in‐plane orientation is slightly higher than that of the sample printed in the cross‐plane orientation, which is likely due to the stronger intralayer bonding than the interlayer bonding.^[^ ^24^ ^]^

### 2.5 Power‐Generating Performance of the 3D‐Printed TEGs

**Figure 4:**
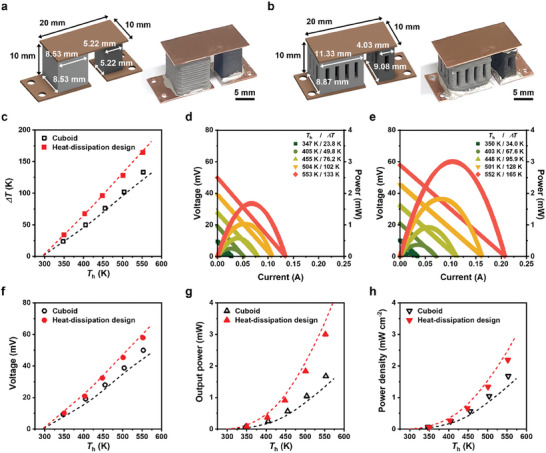
Power‐generating TEG with 3D‐printed heat‐dissipation‐designed TE legs. Photographs and Illustrations of TEGs consisted of a) cuboid and b) heat‐dissipation‐designed ternary Ag chalcogenide TE legs. c) Temperature differences of TEGs consisted of cuboid and heat‐dissipation‐designed TE legs under differing hot side temperatures. Current‐dependent output voltage and power of TEG consisted of d) cuboid and e) heat‐dissipation‐designed TE legs under differing hot side temperatures. f) Voltages, g) output power, and h) power density of TEGs consisted of cuboid and heat‐dissipation‐designed legs under differing hot side temperatures. The points and dashed lines are the measured values and the simulated trend calculated by the FEM method, respectively.

To verify the effectiveness of the heat‐dissipation design, we fabricated a TEG consisting of p‐type AgSbTe_2_ and n‐type AgBiSe_2_ TE legs with optimal designs (**Figure** **4**a,b). Specifically, the device was constructed with the twenty‐pin p‐type leg and eight‐pin n‐type leg, in which the cross–sectional area ratio of the p‐type and n‐type legs was 2.5. As a control sample, the TEG with cuboids of p‐type AgSbTe_2_ and n‐type AgBiSe_2_ with the same volume and cross–sectional area ratio was fabricated. The 3D‐printed TE legs were connected to a Cu plate, which served as an electrode, using Ag adhesive solders. The dimensions of the electrodes were set to be the same for both types of TEG to ensure that the convective cooling through the cold‐side electrodes was identical. To evaluate the power‐generating performances of the TEGs, they were mounted on a hot reservoir that maintained a constant hot‐side temperature. The cold side was cooled by natural convection in an N_2_‐filled chamber to observe the self‐heat‐dissipation effect from the designed TE legs without any active cooling components, such as a heat sink or water‐circulating coolant. Under steady‐state conditions, we comparatively measured the cold‐side temperature, output voltage, and module resistance of the heat‐dissipating and cuboid TEGs. With increasing hot‐side temperature, the measured temperature differences increased for both TEGs and were in good agreement with the simulation results. Moreover, over the entire temperature range of the hot side (300–550 K), the cold‐side temperatures of the heat‐dissipating TEG were lower than those of the cuboid TEG. At the hot‐side temperature of 550 K, the maximum Δ*T* reached 165 K in the heat‐dissipating TEG, which was ≈25% higher than that obtained in the cuboid TEG (Figure 4c). These higher Δ*T* created in the heat‐dissipating TEG were clearly reflected in its output voltages, which were higher than those obtained in the cuboid TEG in the entire temperature range. In addition, both the TEGs showed almost linear increases in the output voltages and quadratic increases in the output power, demonstrating the reliability of the measurements (Figure 4d,e). Furthermore, the maximum output voltage of 57.9 mV and output power of 3.00 mW under the maximum Δ*T* of 165 K was achieved, which was ≈16% and ≈80% higher than that obtained in the cuboid TEG, respectively, under the same condition (Figure 4f,g). Consequently, the heat‐dissipating TEG exhibited the maximum power density of 2.19 mW cm^−2^, which was ≈31% higher than that obtained in the cuboid TEG (Figure 4h). These results clearly demonstrate the feasibility of the heat‐dissipation design in TEGs for maximizing power performance and system compactification.

## 3 Conclusion

We have demonstrated the thermal design strategy and 3D printing realization of heat‐sink‐integrated heat‐dissipating TE legs, which exhibited self‐and efficient heat‐dissipation properties of TE legs without any heavy active cooling component in the power‐generating system. 3D FEM modeling was developed for the heat‐dissipation design and the optimal cross–sectional area ratio of the n‐type TE leg and p‐type TE legs to maximize the temperature difference within the TEG and the resulting output power. The pure AgBiSe_2_ and AgSbTe_2_ particle‐based colloid inks without any organic additives exhibited high viscoelasticity due to the intrinsic surface charge of the TE particles, exhibiting excellent 3D printability to realize complex 3D TE architectures such as vertical filaments, lattices, and bridges with high *ZT* values of 0.49 at 700 K for AgBiSe_2_ and 1.20 at 600 K for AgSbTe_2_. The 3D printing fabrication and evaluation of the designed heat‐dissipating TE legs and their power‐generating devices clearly demonstrate the feasibility of our design strategy for maximizing the heat‐dissipation effect in TE legs. Our thermal design strategy, which is characterized by its simplicity, can serve as a foundation that can be extended or integrated with state‐of‐the‐art thermal design tools. In addition, our work can contribute to a broader range of thermal management solutions by extending the applicability of our approach to electronic and energy devices as well as providing versatility that can be applied to other TE materials. Ultimately, this ease of system integration could accelerate the application of TE technology in everyday life using thermally designed TE devices.

## 4 Experimental Section

### Materials

Glycerol (>99.5%) was purchased from Sigma Aldrich. Powder of Ag (99.9%) was purchased from Alfa Aesar. Elemental granules of Bi (99.999%), Sb (99.999%), Se (99.999%), and Te (99.999%) were purchased from 5N Plus. All the elements and chemicals were used without additional purification.

### Synthesis of AgBiSe_2_ and AgSbTe_2_‐based 3D Printing Inks

All the synthesis steps were conducted under an N_2_ atmosphere. The n‐type powders of AgBiSe_2_ and p‐type powders of AgSbTe_2_ were prepared by mechanical alloying using a planetary ball milling process (Planetary Mono Mill, PULVERISETTE 6) with 450 rpm for 11 h. The ball milling process was conducted using a zirconia jar and zirconia balls with a diameter of 5 mm. The ball‐to‐powder weight ratio was fixed at 5:1. The ball‐milled powders were sieved under 45 µm to ensure the small particle size and the particle size distribution. The particle size, size distribution, and ζ‐potential data were measured by using electrophoretic light scattering (ELS) analysis with a Nano‐ZS of Malvern instrument. The particle dispersions for ELS analysis were prepared by dispersing AgBiSe_2_ and AgSbTe_2_ particles in the polar medium of N‐methyl formamide (NMF) through ultrasonication. The *ζ*‐potential data was measured by using electrophoretic light scattering (ELS) analysis with a Nano‐ZS of Malvern instrument. The AgBiSe_2_ and AgSbTe_2_ inks were prepared by mixing 15 g of ball‐milled AgBiSe_2_ and 10 g of ball‐milled AgSbTe_2_ powders, respectively, with 5 g of glycerol using a planetary centrifugal mixer (ARM‐100, Thinky). The mixing process was performed by repeating three times for a total of 5 min.

### Rheological Properties of the 3D Printing Inks

The rheological properties of AgBiSe_2_ and AgSbTe_2_ inks were analyzed by a rotational rheometer (Haake MARS III, Thermo Scientific) equipped with a coaxial cylinder geometry. The frequency sweep tests were carried out at a constant stress of 1 Pa. The stress sweep tests and the three‐interval thixotropy tests (3ITTs) were conducted over the range of 0.005–300 Pa and at various stresses (1, 5, 10, 50, and 200 Pa), respectively, at a frequency of 1 rad s^−1^. All measurements were conducted at room temperature.

### 3D Printing and Heat Treatment

The 3D printing process was conducted using a home‐built extrusion‐based 3D printer. All the processes were performed at the appropriate nozzle diameters, printing speeds, and printing pressures for the sliced CAD drawings. Synthesized TE inks were contained in syringes (Saejong) and deposited onto graphite substrates using a pneumatic pressure controller. All the 3D‐printed samples were dried for over 12 h at temperatures above 413 K to fully evaporate glycerol. The sintering processes were conducted at 988 K for 1 to 5 h in the case of AgBiSe_2_ and 803 K for 1 to 9 h in the case of AgSbTe_2_ samples. All the heat treatments were conducted under the N_2_ atmosphere.

### Characterization of 3D‐printed Samples

The XRD patterns were obtained using X'pert Pro (PANalytical) with a Cu Kα X‐ray source (wavelength of 1.5418 nm) equipped with an X'Celerator detector, operating at 40 kV and 200 mA. The OM images were obtained using an Olympus BX51M. The SEM images were obtained using a field‐effect SEM (FEI Nova NanoSEM 230) operated at 10 kV. The XPS measurement of the particles was conducted using an X‐ray photoelectron spectrometer (K‐Alpha, Thermo Scientific). The compressive tests were performed using universal testing systems (5948 MicroTester, Instron) with a strain rate of 1 × 10^−3^ s^−1^.

### TE Properties of 3D‐printed Samples

The temperature‐dependent electrical conductivity and Seebeck coefficient were measured using a thermal analyzer (SBA 458 Nemesis, Netzsch) in the temperature range from 300 to 700 K under an Ar atmosphere. The thermal conductivity (*κ*) was calculated from the relationship of *κ* = *ρC*_p_ *D*, where *ρ* was density, *C*_p_ was specific heat capacity, and *D* was thermal diffusivity. The density of the sintered samples was estimated by measuring their weight and volume after polishing them into a flat cuboid shape, ensuring minimal measurement errors in length, width, and height. The specific heat capacity was calculated using the Dulong–Petit equation. The thermal diffusivity was measured using laser flash analysis (LFA 467HT, Netzsch). The lattice thermal conductivities of 3D‐printed samples with 100% relative density were calculated using effective medium theory, which was shown as

(3) $$\kappa_{L}=\kappa_{h}\frac{2-2\Phi }{2+\Phi }$$

where *κ*_h_ was the lattice thermal conductivity of the host material and *Φ* was porosity. The carrier concentrations and mobilities at room temperature were measured using Hall measuring equipment (HMS‐5000, ECOPIA).

### Simulation of TEG Performance

A 3D finite element model (FEM) was employed via COMSOL Multiphysics to evaluate the performance of the thermoelectric generator (TEG). The FEM approach subdivided a resistor into smaller elements, allowing for a detailed assessment of the electrical characteristics of each element to predict the overall behavior of the structure. This method proved particularly effective for analyzing the electrical properties of resistors with complex 3D geometries, offering more precise calculations of electrical resistance, especially in resistors with irregular shapes or intricate designs, compared to traditional calculations.^[^ ^25^ ^]^ In the simulation, the designed module, thermoelectric module, heat transfer module, and AC/DC module of COMSOL were used to calculate the temperature distribution in a TEG with resulting electrical outputs. The temperature distribution and electrical potential under the steady‐state condition were obtained by following non‐linear coupled equations:

(4) $$-\nabla \left(\left(\sigma S^{2}T+k\right)\nabla T\right)-\nabla \left(\sigma ST\nabla V\right)=\sigma \left({\left(\nabla V\right)}^{2}+S\nabla T\nabla V\right)$$

(5) $$\nabla \left(\sigma \nabla V\right)+\nabla \left(\sigma S\nabla T\right)$$

where *T*, *V*, *S*, *k*, and *σ* were the temperature, the electrical potential, the Seebeck coefficient, the thermal conductivity, and the electrical conductivity, respectively. The electrical resistance was calculated by dividing the potential (*V*_oc_) by the short‐circuit current (*I*_sc_), providing a practical approach for determining the electrical resistance of a TEG under operational conditions.^[^ ^9^, ^26^ ^]^ The maximum output power (*P*_max_) was calculated using *P_max_* = *V*_oc_^2^/4*R*. The employed material properties were interpolated with cubic spline based on the measurements of 3D‐printed ternary Ag chalcogenide materials, as depicted in Figure 3. All the simulated models comprised top and bottom electrodes, between which the TE leg was sandwiched. The electrodes had dimensions of 10 × 10 × 0.3 mm^3^ and were the same for all models for a fair comparison. The differing geometries of the TE legs—including cuboid, 1‐, 4‐, and 16‐pin models—were designed with a uniform height of 10 mm and a volume of 500 mm^3^. For heat dissipation‐designed TE legs, the TE pins were sandwiched with 2.5 mm of TE materials thickness to minimize contact resistance. As boundary conditions for the single TE leg model, the top side of the upper electrode was electrically grounded, and all other sides were electrically insulated. In the uni‐couple of the TEG, the cold‐side electrode of the p‐type TE leg was grounded, with the electrical load directly connected to the cold‐side electrodes. The temperature of the top‐side electrode was maintained at a fixed temperature of 573 K, whereas all other surfaces were subjected to convection conditions. It was assumed that the convection coefficient for all surfaces was consistent, set at 5 W m^−2^ K^−1^, with an ambient temperature of 25 °C. The optimization of the cross–sectional area of p‐type and n‐type TE legs was conducted at the constant total volumes of n‐type and p‐type legs. In this computation, it was assumed that the optimum cross–sectional area ratio was similar for both the cuboid and heat‐dissipation designs. Based on the optimization, an optimum device for the heat‐dissipation model was designed, which had a 20‐pin p‐type leg and an eight‐pin n‐type leg. The dimension of the optimum device is depicted in Figure 4A,B. The simulation to compare the experimental measurement of TEG included the contact resistance between the electrodes and TE legs. The contact resistivity was calculated based on the measurement of the area‐dependent contact resistances (Figure S28, Supporting Information). The employed material properties were based on the measurements of 3D‐printed ternary Ag chalcogenide materials, as shown in Figure 3. All of the simulated models, including the cuboid, 1‐, 4‐, and 16‐pin models, had a uniform height of 10 mm and volume of 500 mm^3^ for each TE leg. For heat dissipation‐designed TE legs, the TE pins were sandwiched with 2.5 mm of TE materials thickness to reduce contact resistance. As the boundary conditions, the top side of the upper electrode was set to a fixed temperature of 573 K and electrically grounded while all other surfaces were subjected to a convection condition with a convection coefficient of 5 W m^−2^ K^−1^ and an ambient temperature of 25 °C. The optimization of the cross–sectional area of p‐type and n‐type TE legs was conducted at the constant total volumes of n‐type and p‐type legs. The simulation to compare the experimental measurement of TEG included the contact resistance between the electrodes and TE legs.

### Fabrication and Evaluation of TEGs

TEGs were fabricated by integrating with Cu electrodes using Ag epoxy (EPOXIOHM EO‐98HT, EpoxySet Inc.) for the n‐type AgBiSe_2_ leg and Ag paste (Pyro‐Duct 597‐A, Aremco) for the p‐type AgSbTe_2_ leg, respectively. The output voltage, resistance, and output power of the TEGs were measured onto a ceramic heater with the size of 70 × 15 mm^2^ as a heat source. The ceramic heater generated heat by controlling voltage through the DC power supply. The hot‐side and cold‐side temperatures were measured by using a Keithley 2000 multimeter with K‐type thermocouples. The whole measurement was conducted in a vacuum chamber under natural convection of the N_2_ atmosphere.

## Conflict of Interest

The authors declare no conflict of interest.

## Author Contributions

K.K., S.C., and J.L. contributed equally to this work. K.K., S.C., J.L., and J.S.S. designed the experiments, analyzed the data, and wrote the paper. K.K., S.C., H.J., S.‐H.L., S.B. J.‐Y.K., and H.G.C., carried out the synthesis and basic characterization of materials. K.K. and S.C. performed the printing of metal chalcogenides. K.K., S.‐h.J., S.J., and K.T.K. performed the characterization of thermoelectric properties. J.L. conducted the numerical simulation of thermoelectric materials and devices. K.K. and S.C. fabricated and evaluated the thermoelectric devices. All authors discussed the results and commented on the manuscript.

## Supporting information

Supporting Information

Supplemental Video 1

## Data Availability

The data that support the findings of this study are available from the corresponding author upon reasonable request.

## References

[1] a) F. J. DiSalvo , Science 1999, 285, 703; 10426986 10.1126/science.285.5428.703

[2] a) S. Lohrasbi , R. Hammer , W. Essl , G. Reiss , S. Defregger , W. Sanz , IEEE Access 2020, 8, 166880;

[3] a) H. S. Han , S. Y. Kim , T. H. Ji , Y. J. Jee , D. Lee , K. S. Jang , D. H. Oh , presented at 2008 11th Intersoc. Conf. Thermal Thermomech. Phenomena Electronic Systems 2008;

[4] a) M. He , Y. Zhao , B. Wang , Q. Xi , J. Zhou , Z. Liang , Small 2015, 11, 5889; 26448629 10.1002/smll.201502153

[5] a) F. Kim , B. Kwon , Y. Eom , J. E. Lee , S. Park , S. Jo , S. H. Park , B. S. Kim , H. J. Im , M. H. Lee , T. S. Min , K. T. Kim , H. G. Chae , W. P. King , J. S. Son , Nat. Energy 2018, 3, 301;

[6] a) S. N. Guin , A. Chatterjee , D. S. Negi , R. Datta , K. Biswas , Energy Environ. Sci. 2013, 6, 2603;

[7] a) M. D. Nielsen , V. Ozolins , J. P. Heremans , Energy Environ. Sci. 2013, 6, 570;

[8] a) H. Wang , J. F. Li , M. Zou , T. Sui , Appl. Phys. Lett. 2008, 93, 202106;

[9] B. Şişik , S. LeBlanc , Front. Mater. 2020, 7, 595955.

[10] Y. G. Lee , J. Kim , M. S. Kang , S. H. Baek , S. K. Kim , S. M. Lee , J. Lee , D. B. Hyun , B. K. Ju , S. E. Moon , J. S. Kim , B. Kwon , Adv. Mater. Technol. 2017, 2, 1600292.

[11] K. Sun , T. S. Wei , B. Y. Ahn , J. Y. Seo , S. J. Dillon , J. A. Lewis , Adv. Mater. 2013, 25, 4539. 23776158 10.1002/adma.201301036

[12] Y. Eom , F. Kim , S. E. Yang , J. S. Son , H. G. Chae , J. Rheol. 2019, 63, 291.

[13] a) N. Su , P. Zhu , Y. Pan , F. Li , B. Li , Energy 2020, 195, 116892;

[14] a) T. Jain , Y. M. Tseng , C. Tantisuwanno , J. Menefee , A. Shahrokhian , I. Isayeva , A. Joy , ACS Appl. Polym. Mater. 2021, 3, 6618;

[15] J. H. Wernick , S. Geller , K. E. Benson , J. Phys. Chem. Solids 1958, 7, 240.

[16] H. Jang , M. Y. Toriyama , S. Abbey , B. Frimpong , J. P. Male , G. J. Snyder , Y. S. Jung , M. W. Oh , Adv. Mater. 2022, 34, 2204132. 10.1002/adma.202204132 35944565

[17] a) S. Li , S. Hou , W. Xue , L. Yin , Y. Liu , X. Wang , C. Chen , J. Mao , Q. Zhang , Mater. Today Phys. 2022, 27, 100837;

[18] F. Böcher , S. P. Culver , J. Peilstöcker , K. S. Weldert , W. G. Zeier , Dalton Trans. 2017, 46, 3906. 28265625 10.1039/c7dt00381a

[19] H. Zhu , T. Zhao , B. Zhang , Z. An , S. Mao , G. Wang , X. Han , X. Lu , J. Zhang , X. Zhou , Adv. Energy Mater. 2021, 11, 2003304.

[20] J. D. Sugar , D. L. Medlin , J. Alloys Compd. 2009, 478, 75.

[21] a) X. Liu , D. Jin , X. Liang , Appl. Phys. Lett. 2016, 109, 133901;

[22] a) D. Bao , J. Chen , Y. Yu , W. Liu , L. Huang , G. Han , J. Tang , D. Zhou , L. Yang , Z. G. Chen , Chem. Eng. J. 2020, 388, 124295;

[23] M. M. Al Malki , G. J. Snyder , D. C. Dunand , Int. Mater. Rev. 2023, 68, 1050.

[24] a) R. Zou , Y. Xia , S. Liu , P. Hu , W. Hou , Q. Hu , C. Shan , Composites, Part B 2016, 99, 506;

[25] a) D. S. Burnett , Finite Element Analysis: From Concepts to Applications, Addison‐Wesley Pub. Co., Boston 1987;

[26] H. Mamur , R. Ahıska , Int. J. Renewable Energ. Res. 2014, 4, 128.

