# Supplementary material for: Heat‐Dissipation Design and 3D Printing of Ternary Silver Chalcogenide‐Based Thermoelectric Legs for Enhancing Power Generation Performance
Source: Adv Sci (Weinh). 2024 Jun 10;11(30):2402934. doi: 10.1002/advs.202402934 (PMC11321642; doi:10.1002/advs.202402934)
Supplement: Supplementary file 1 — Supporting Information [file ADVS-11-2402934-s002.pdf]

ADVANCED SCIENCE
Open Access

Supporting Information

for *Adv. Sci.*, DOI 10.1002/advs.202402934

Heat-Dissipation Design and 3D Printing of Ternary Silver Chalcogenide-Based Thermoelectric Legs for Enhancing Power Generation Performance

*Keonkuk Kim, Seungjun Choo, Jungsoo Lee, Hyejin Ju, Soo-ho Jung, Seungki Jo, So-Hyeon Lee, Seongheon Baek, Ju-Young Kim, Kyung Tae Kim, Han Gi Chae and Jae Sung Son**

Supporting Information

Heat-Dissipation Design and 3D Printing of Ternary Silver Chalcogenide-Based Thermoelectric Legs for Enhancing Power Generation Performance

Keonkuk Kim, Seungjun Choo, Jungsoo Lee, Hyejin Ju, Soo-ho Jung, Seungki Jo, Sohyeon Lee, Seongheon Baek, Ju-Young Kim, Kyung Tae Kim, Han Gi Chae, and Jae Sung Son[*]

K. Kim, S. Choo, J. Lee, Prof. J. S. Son
Department of Chemical Engineering, Pohang University of Science and Technology (POSTECH), Pohang 37673, Republic of Korea
E-mail: sonjs@postech.ac.kr

H. Ju, S. Lee, S. Baek, Prof. J.-Y. Kim, Prof. H. G. Chae
Department of Materials Science and Engineering, Ulsan National Institute of Science and Technology (UNIST), Ulsan 44919, Republic of Korea

S. -h. Jung, S. Jo, K. T. Kim
Department of 3D Printing Materials, Korea Institute of Materials Science (KIMS), Changwon 51508, Republic of Korea

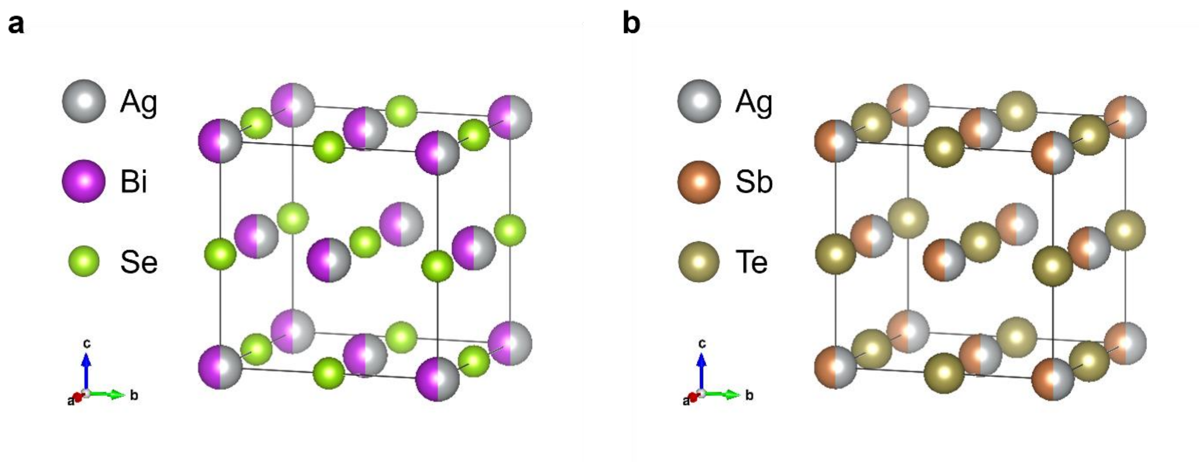

Figure S1. Crystal structure diagrams of cubic phase for a) $AgBiSe_2$ and b) $AgSbTe_2$ visualized using VESTA software.

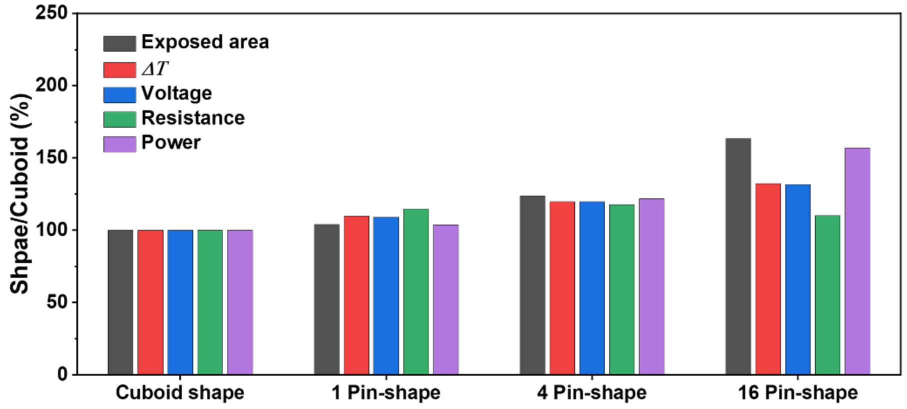

Figure S2. The percentage of exposed surface area, temperature difference, output voltage, electrical resistance, and output power of different shapes compared to those for a cuboid shaped leg using $AgSbTe_2$.

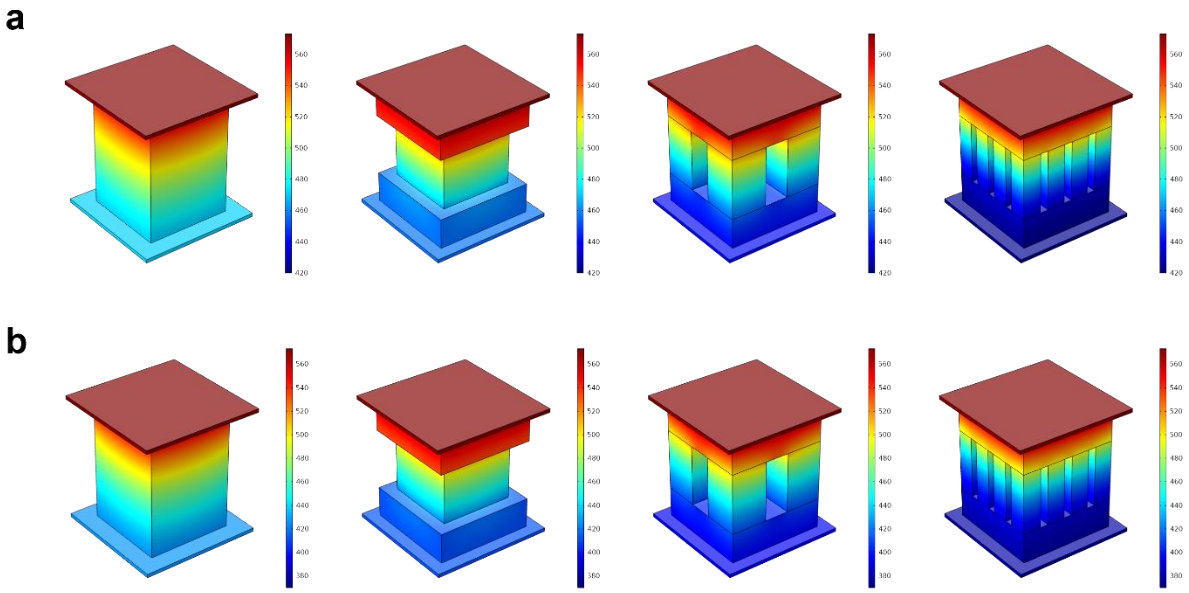

Figure S3. Simulated temperature distributions of cuboid-, 1 pin-, 4 pins- and 16 pins-shaped a) $AgBiSe_2$ and b) $AgSbTe_2$ legs.

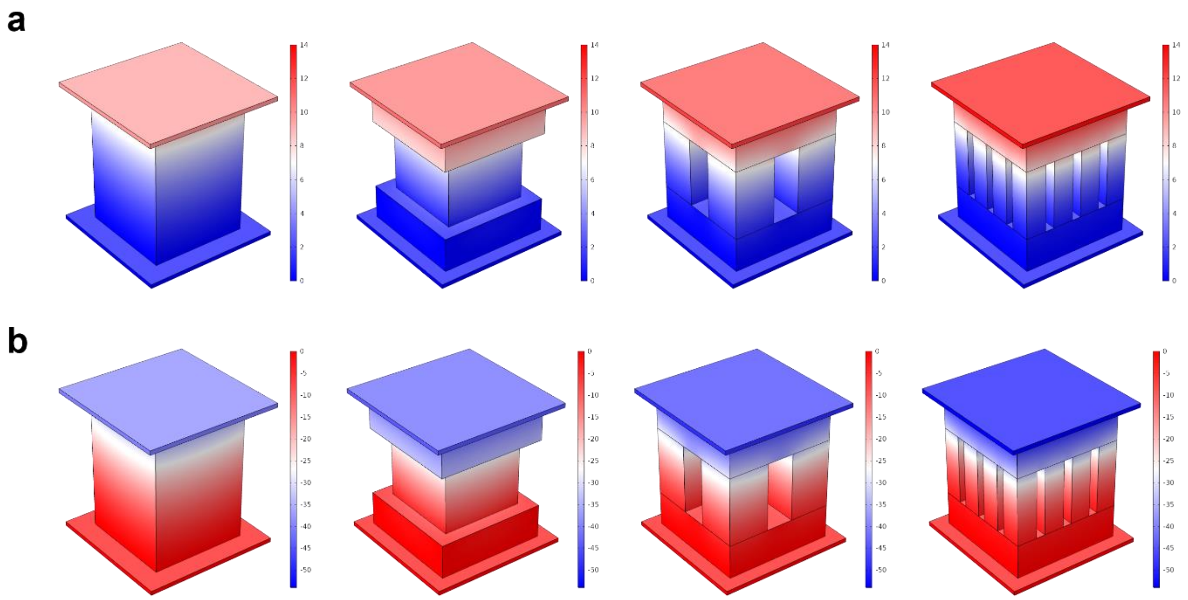

Figure S4. Simulated voltage distributions of cuboid-, 1 pin-, 4 pins- and 16 pins-shaped a) $AgBiSe_2$ and b) $AgSbTe_2$ legs.

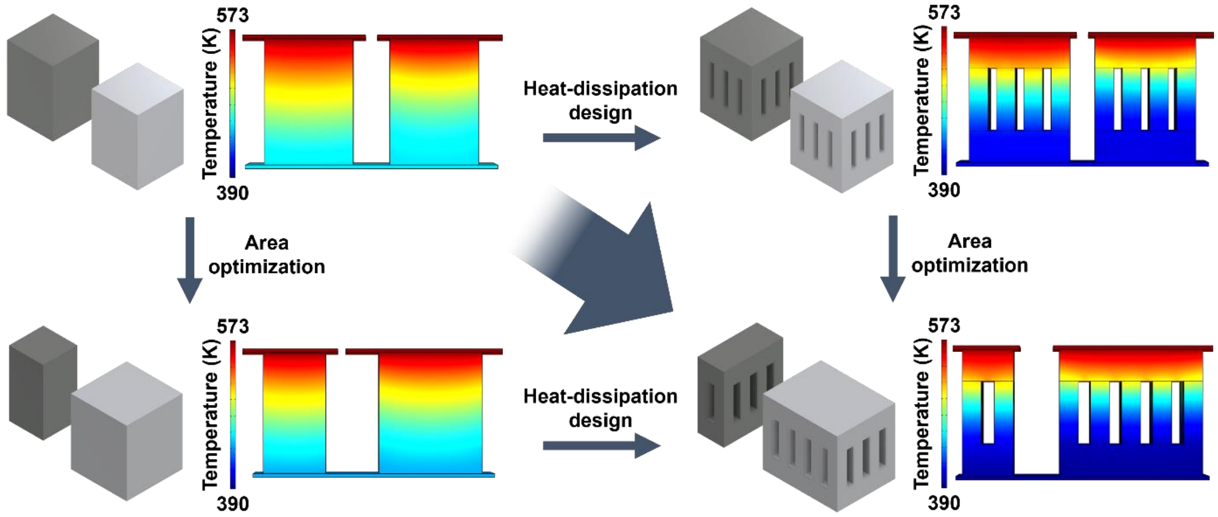

Figure S5. Schematic representation of the optimization process for the TEG shape exhibiting the highest temperature distribution through integration of heat-dissipation design and optimization of n-type and p-type TE leg cross-sectional area ratio.

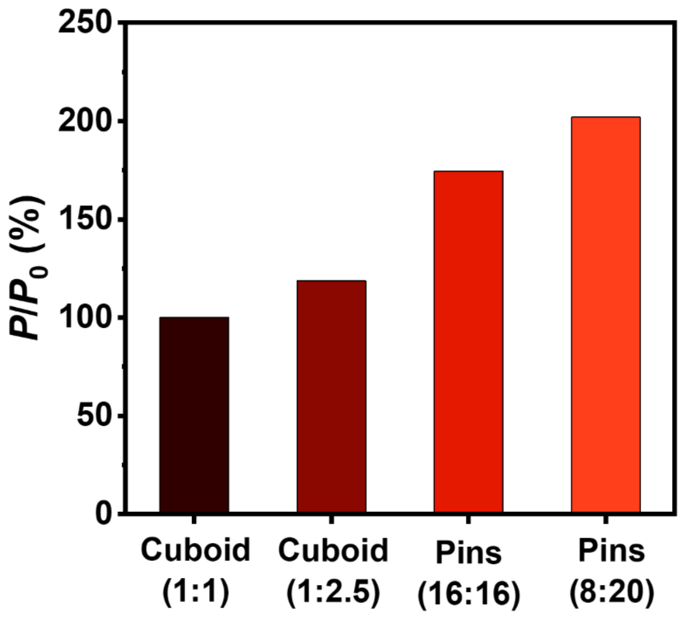

Figure S6. Output power percentage of each TEG shape compared to that of the cuboid design with unoptimized cross-sectional area ratio.

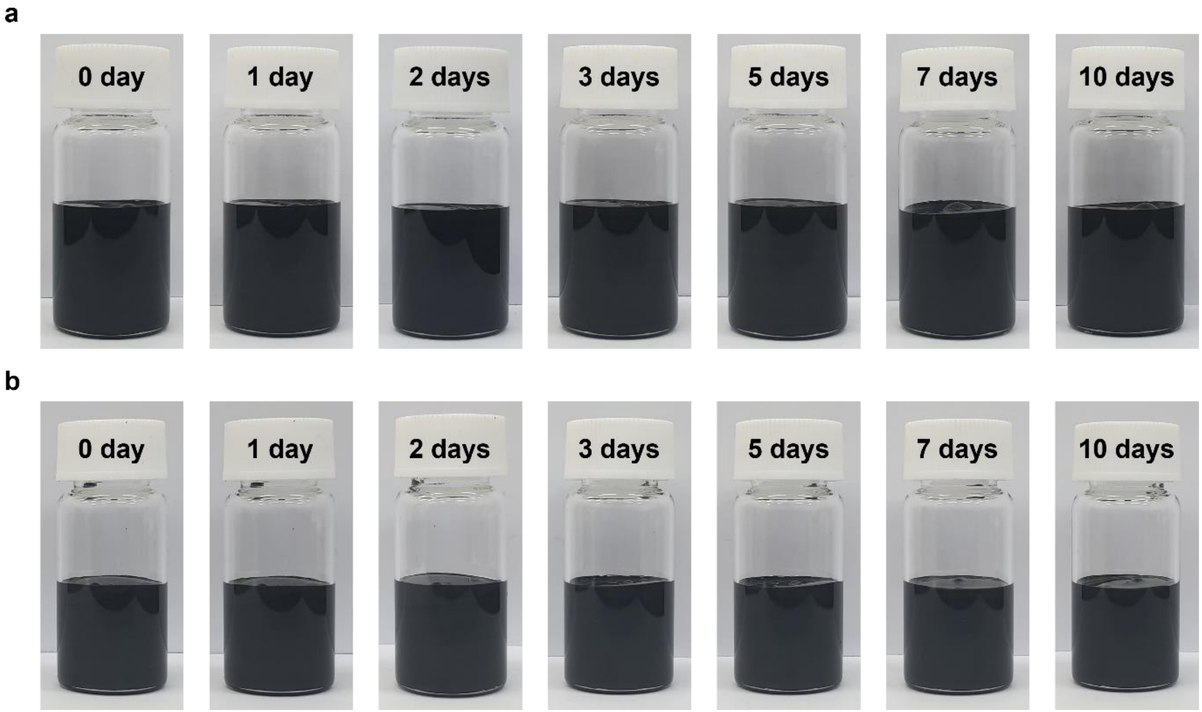

Figure S7. Photographs showing the time-dependent dispersibility of a) $AgBiSe_2$ and b) $AgSbTe_2$ TE inks.

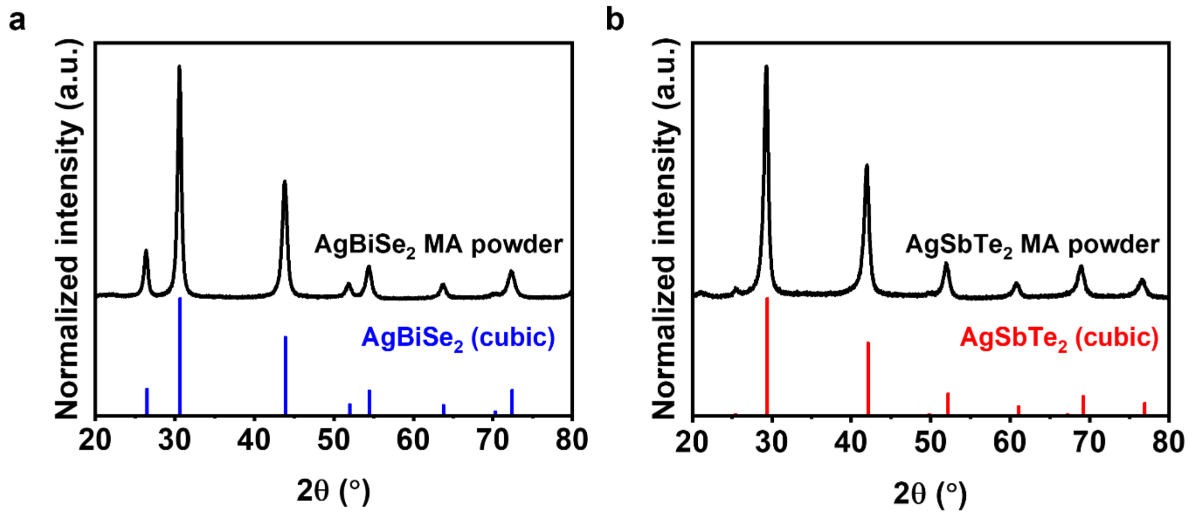

Figure S8. XRD patterns of mechanically alloyed a) $AgBiSe_2$ and b) $AgSbTe_2$ particles.

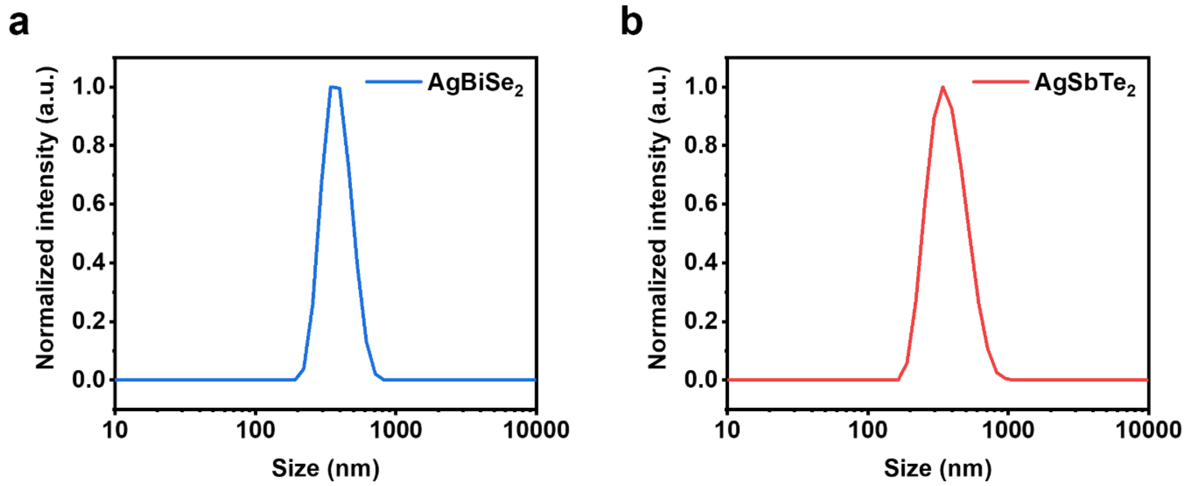

Figure S9. Electrophoretic light scattering (DLS) size of the a) $AgBiSe_2$ and b) $AgSbTe_2$ particles dispersed in NMF.

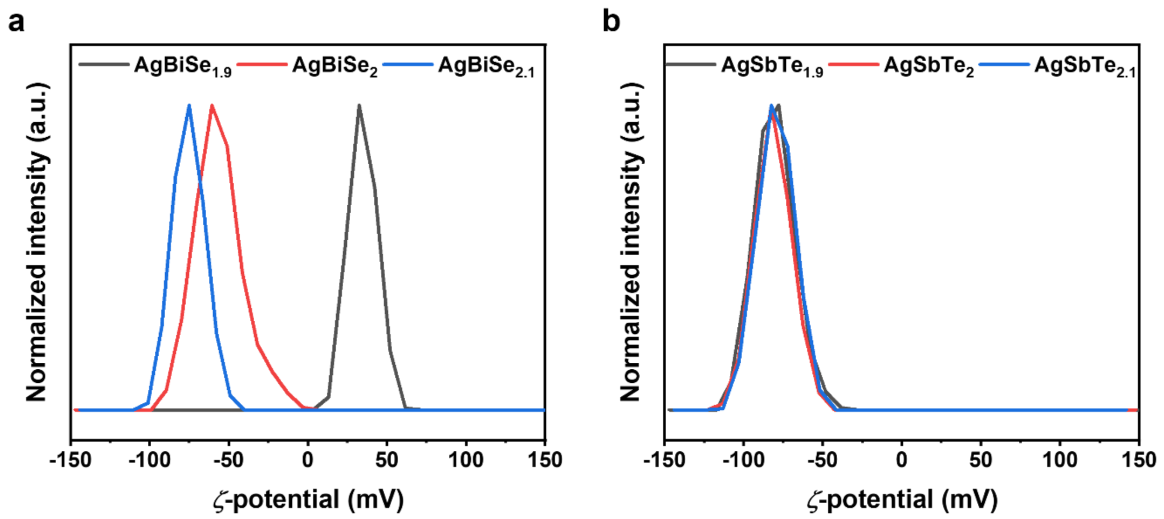

Figure S10. ζ-potential spectrum of a) $AgBiSe_{2+x}$ and b) $AgSbTe_{2+x}$ particles (x=-0.1, 0, 0.1).

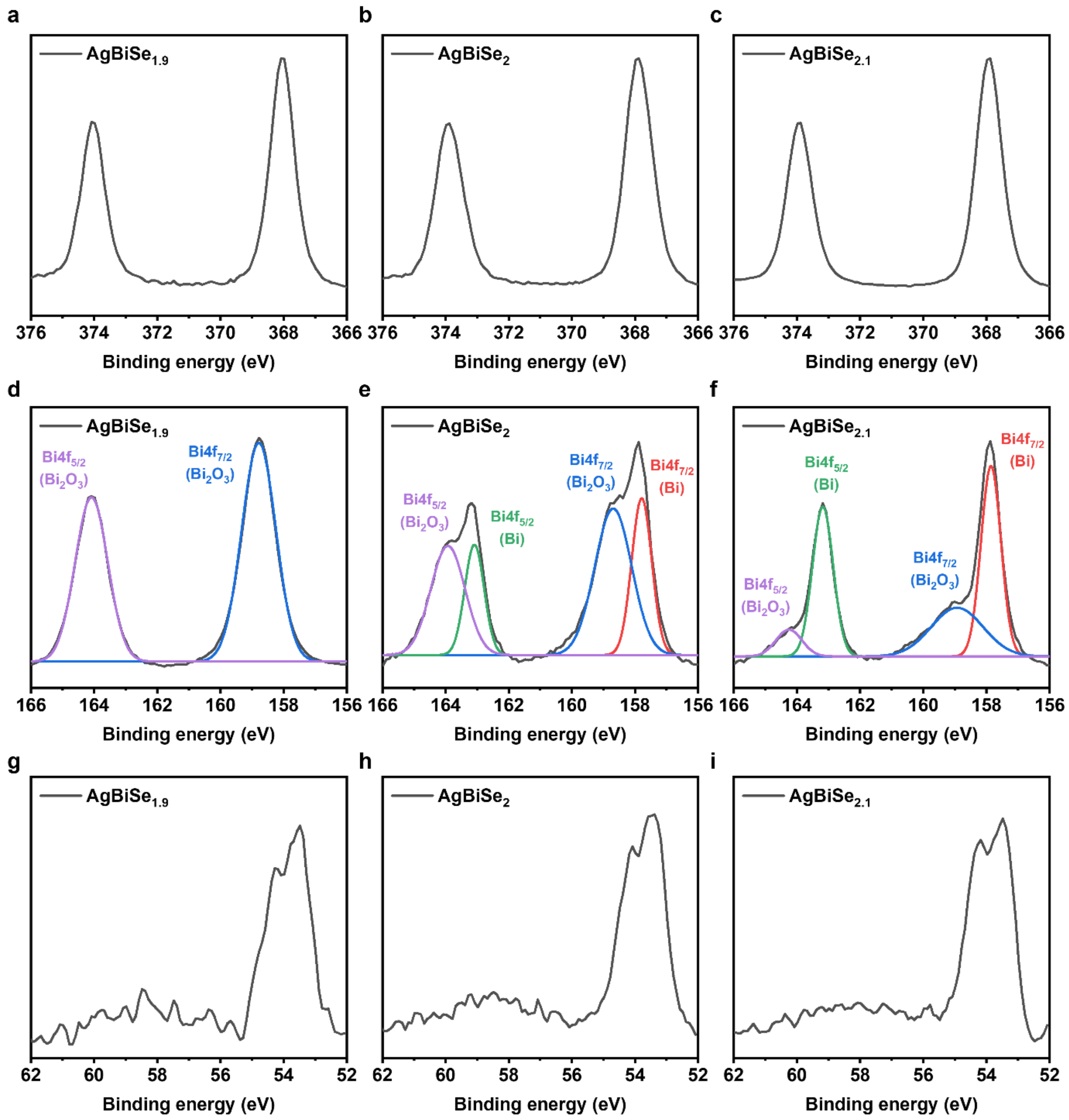

Figure S11. XPS spectra of $AgBiSe_{2+x}$ (x=-0.1, 0, 0.1) particles in the regions of a-c) Ag3d, d-f) Bi4f, and g-i) Se3d.

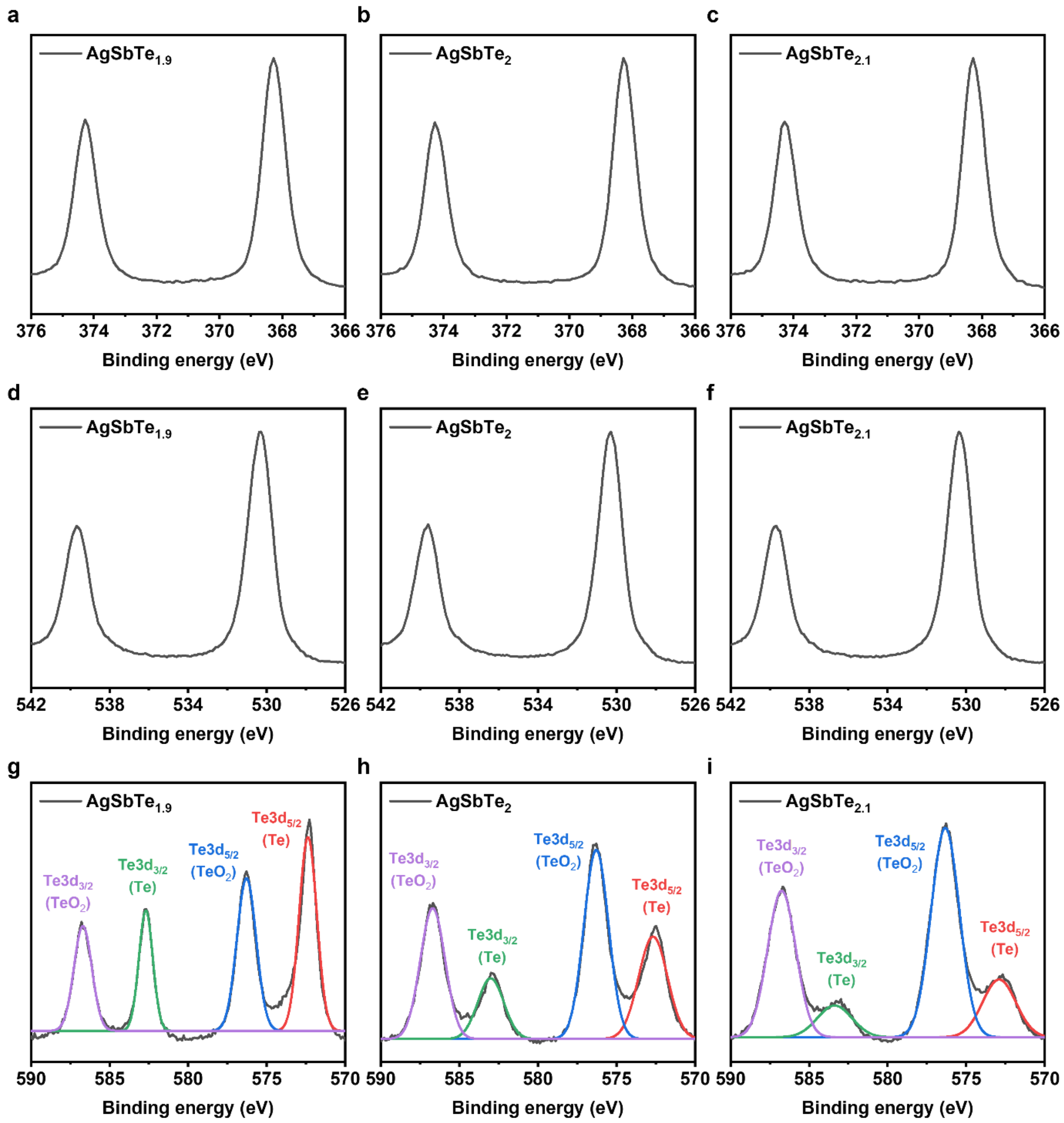

Figure S12. XPS spectra of $AgSbTe_{2+x}$ (x=-0.1, 0, 0.1) particles in the regions of a-c) Ag3d, d-f) Sb3d, and g-i) Te3d.

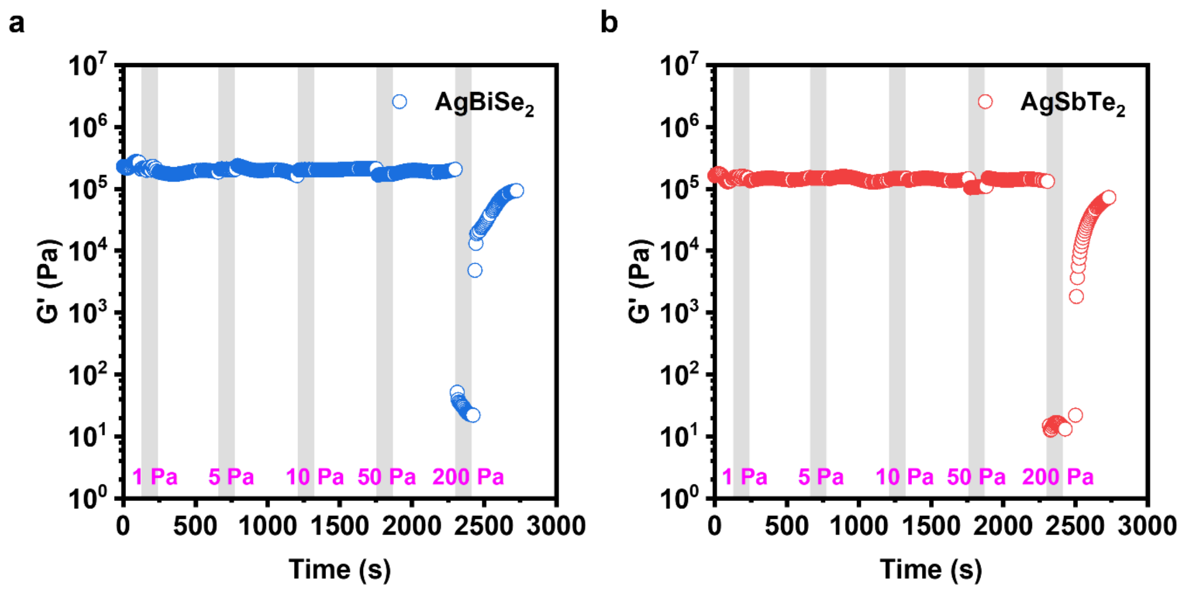

Figure S13. The G' curves from the sequential three-interval thixotropy test (3ITT) of a) $AgBiSe_2$ and b) $AgSbTe_2$ TE inks at various shear stresses (1, 5, 10, 50, and 200 Pa).

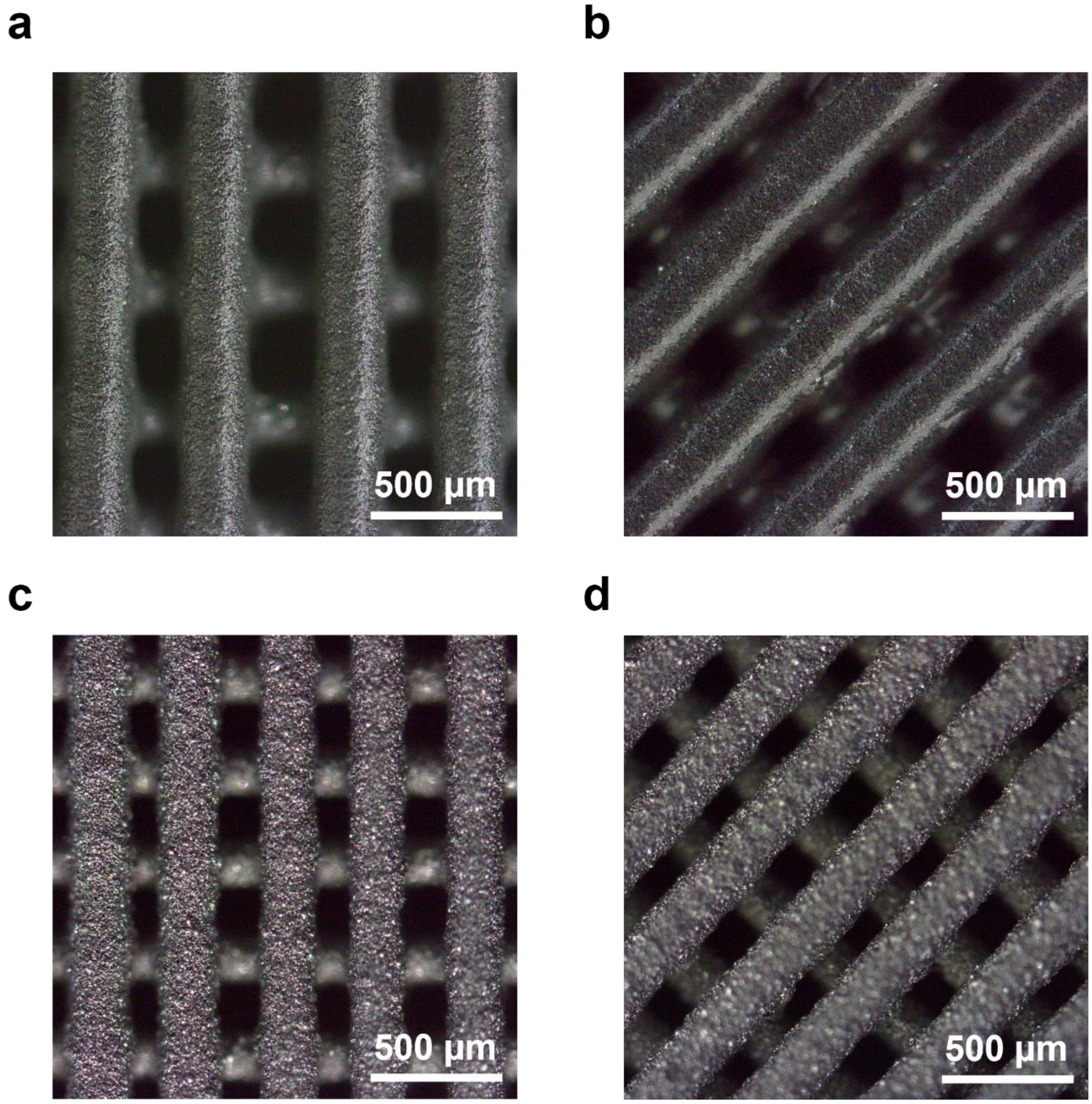

Figure S14. OM images of the a,b) as-printed and c,d) sintered TE filaments.

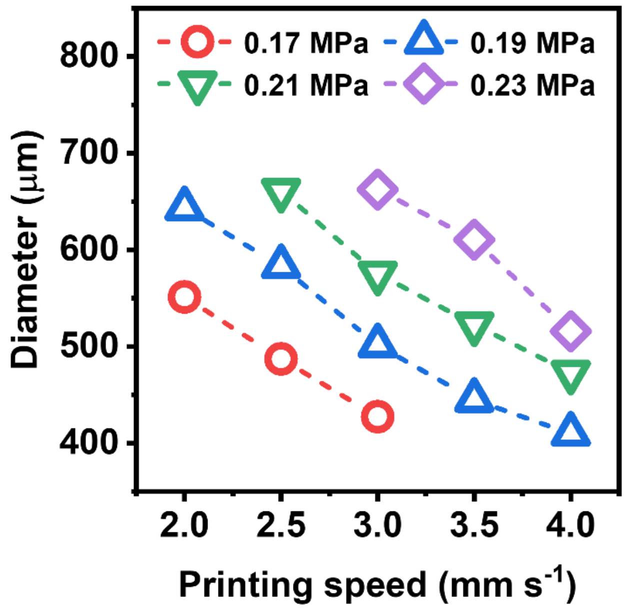

Figure S15. Diameters of the 3D-printed TE filaments versus printing speed for different dispensing pressures using a nozzle with an inner diameter of 510 μm.

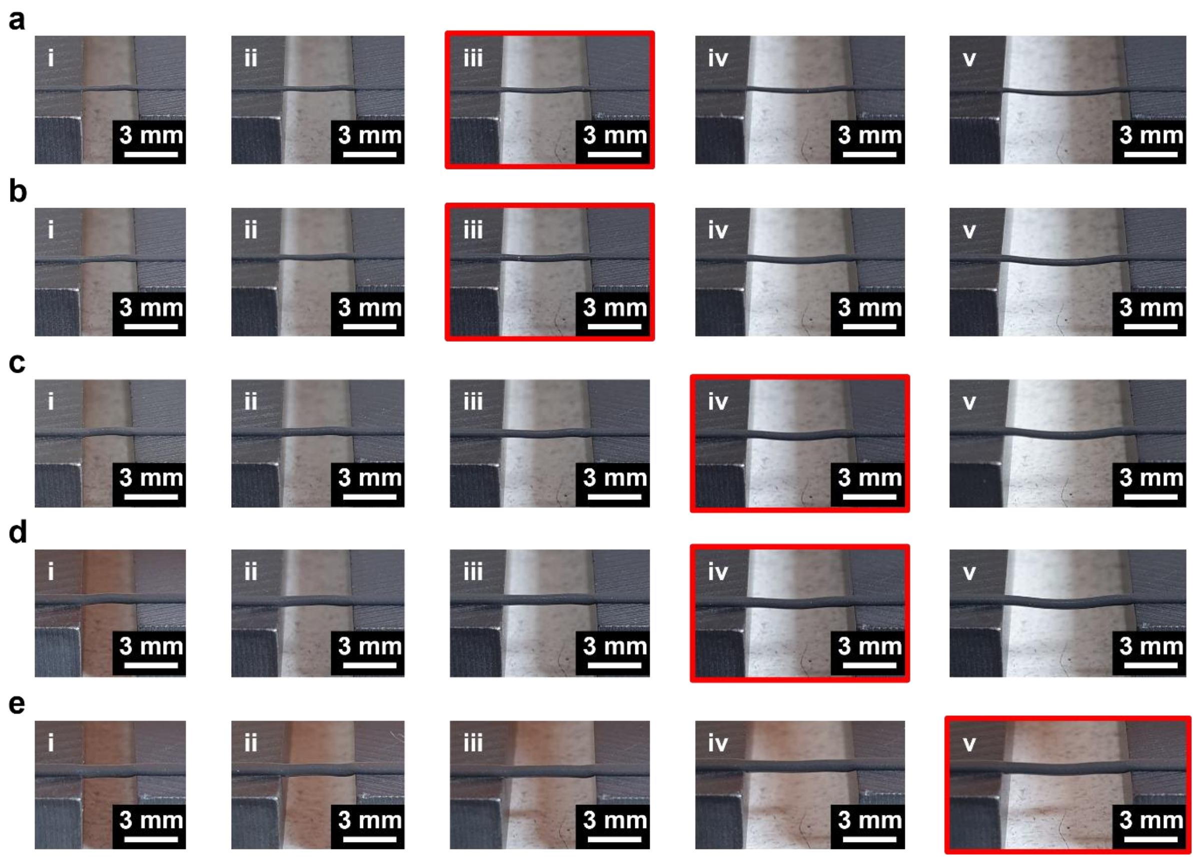

Figure S16. Photographs of bridging TE filaments dependent on the diameters of the filaments of a) 240 µm, b) 340 µm, c) 410 µm, d) 510 µm and e) 610 µm and block-to-block distance between two graphite blocks of (i) 3 mm, (ii) 4 mm, (iii) 5 mm, (iv) 6 mm and (v) 7 mm. The red lines indicate the filaments that start to sag.

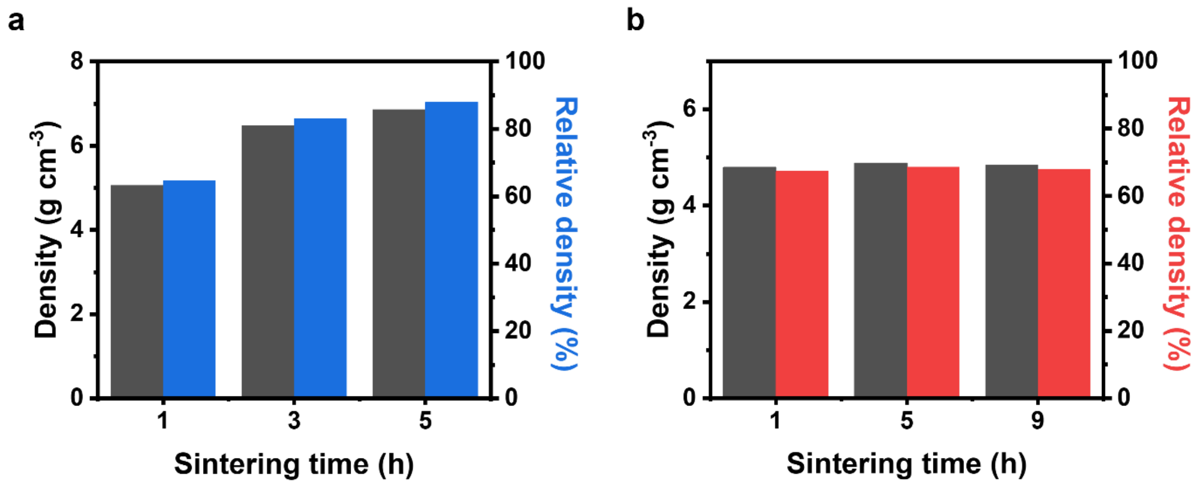

Figure S17. Densities and relative densities of the 3D-printed a) $AgBiSe_2$ and b) $AgSbTe_2$ samples depending on the sintering time.

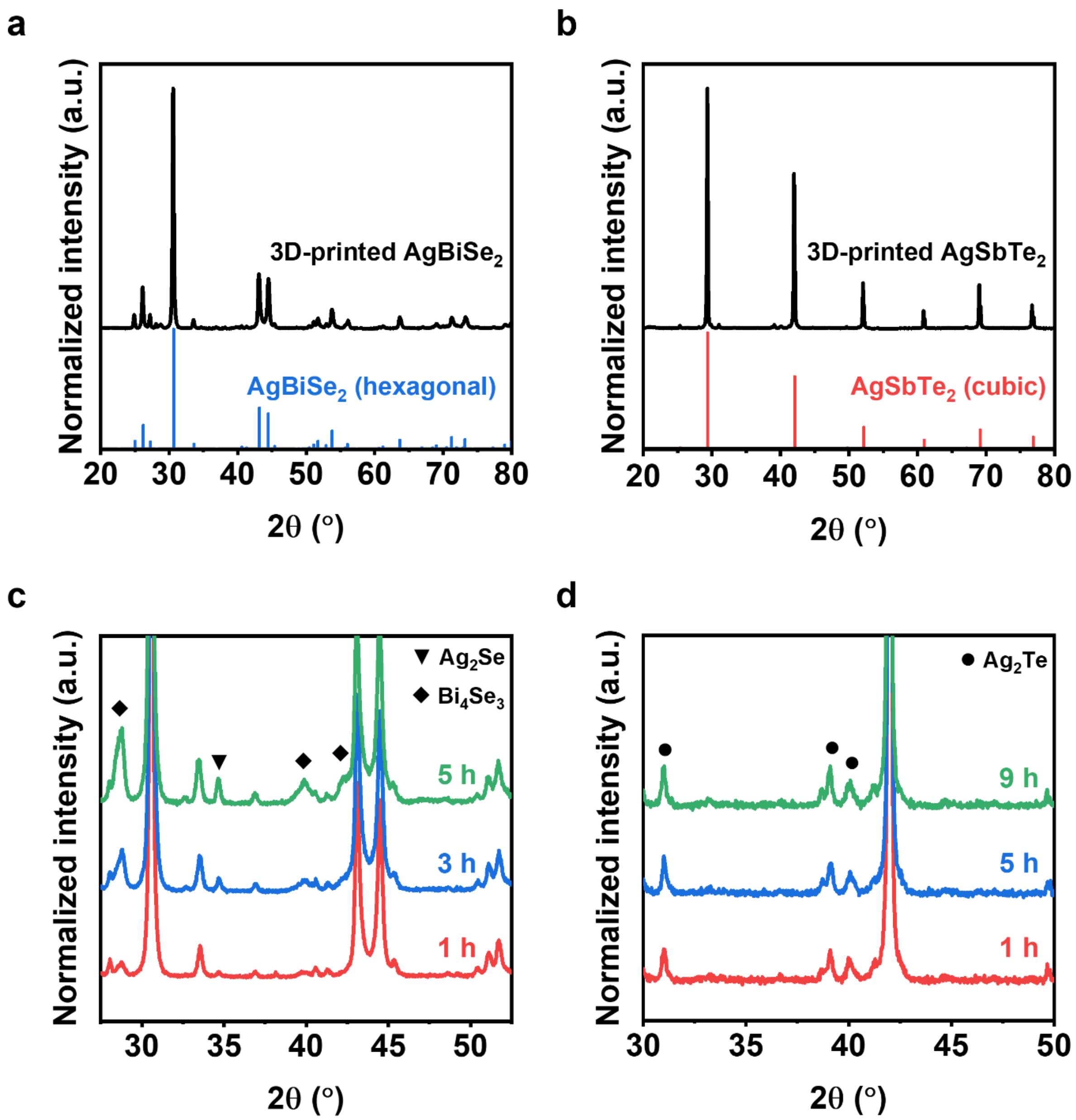

Figure S18. XRD patterns of 3D-printed a) $AgBiSe_2$ and b) $AgSbTe_2$ samples. Enlarged XRD patterns of 3D-printed c) $AgBiSe_2$ and d) $AgSbTe_2$ samples depending on the sintering time.

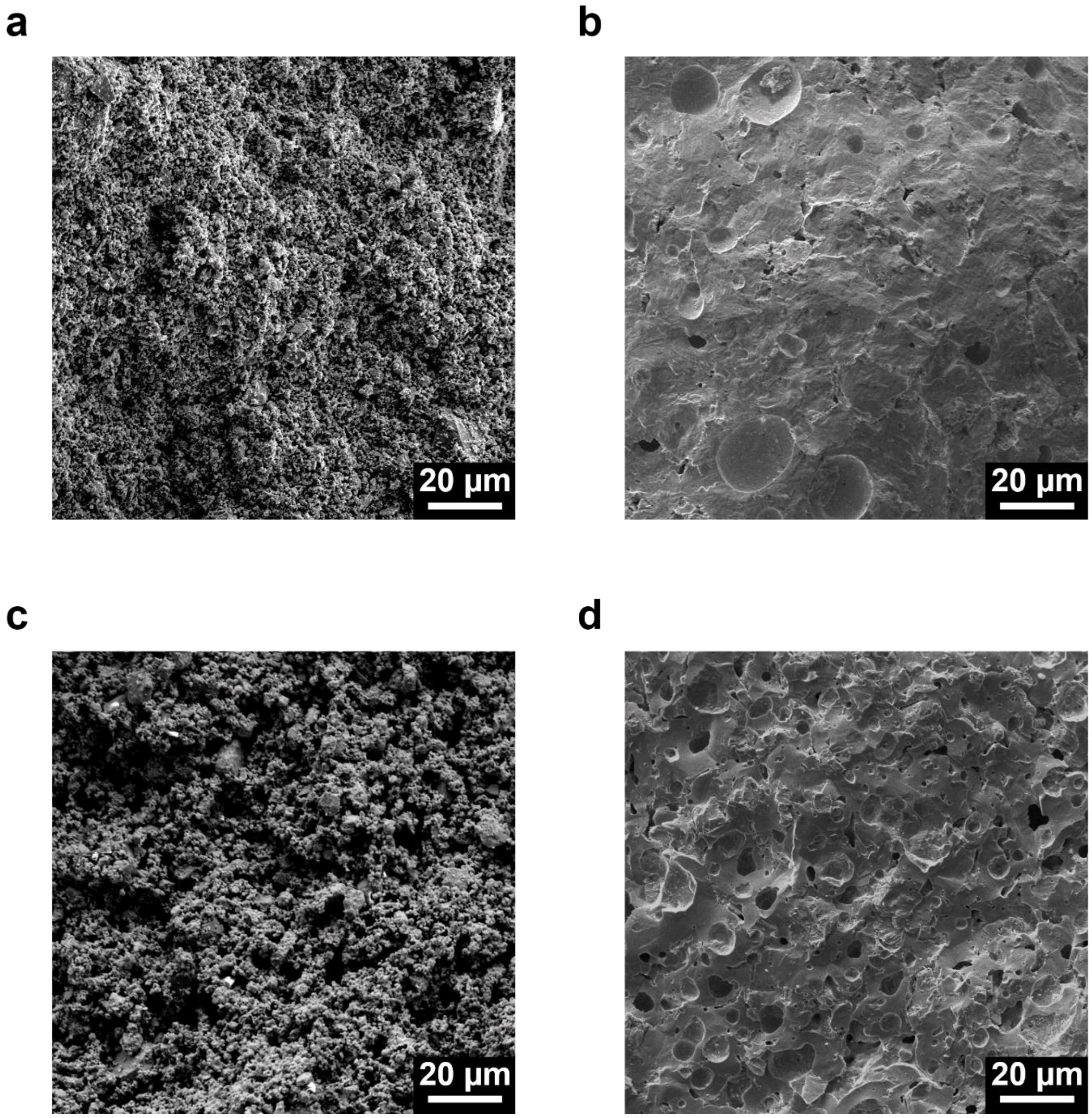

Figure S19. SEM images of a) 3D-printed and b) sintered $AgBiSe_2$ samples and c) 3D-printed and d) sintered $AgSbTe_2$ samples.

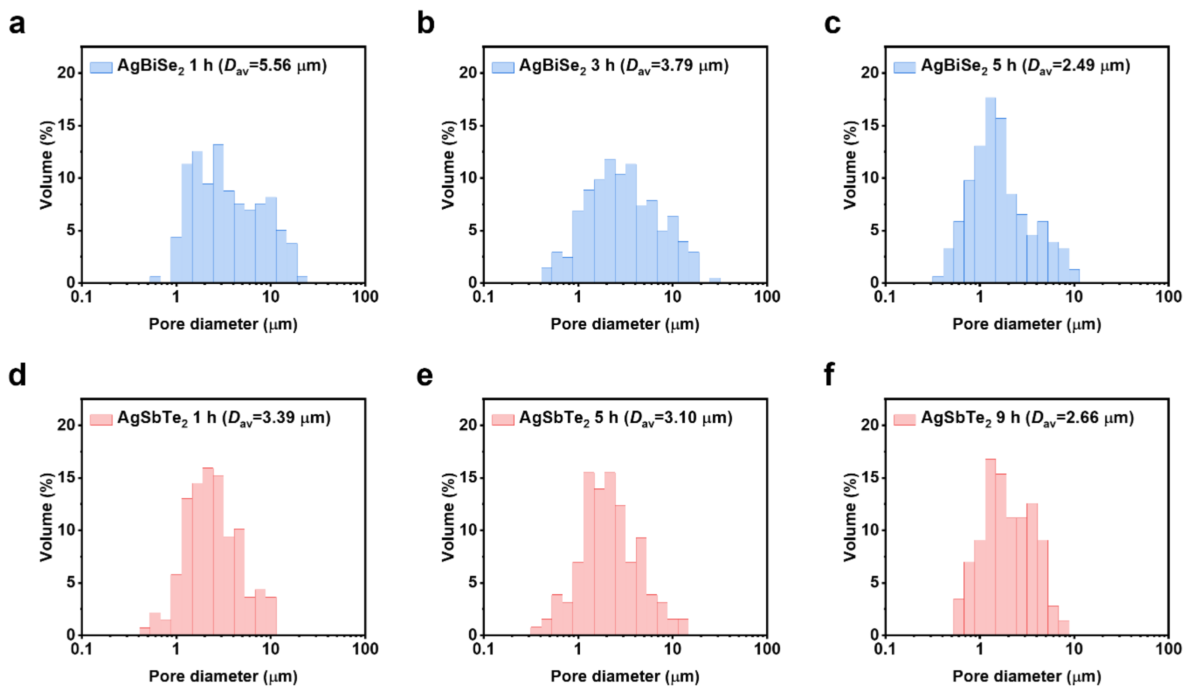

Figure S20. Pore size distribution of 3D-printed a) ABS1, b) ABS3, c) ABS5, d) AST1, e) AST5, and f) AST9 samples. D_{av} denotes the average pore size of each sintered sample.

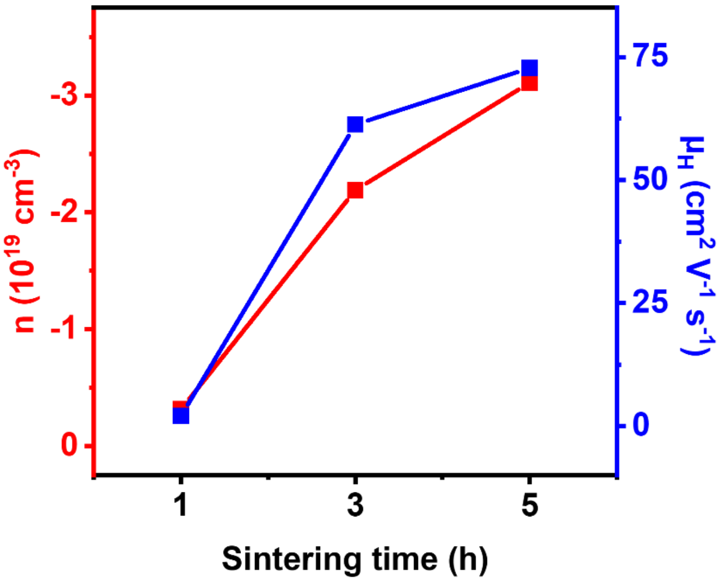

Figure S21. Carrier concentrations and mobilities of the 3D-printed $AgBiSe_2$ samples at room-temperature sintered for 1, 3, and 5 h.

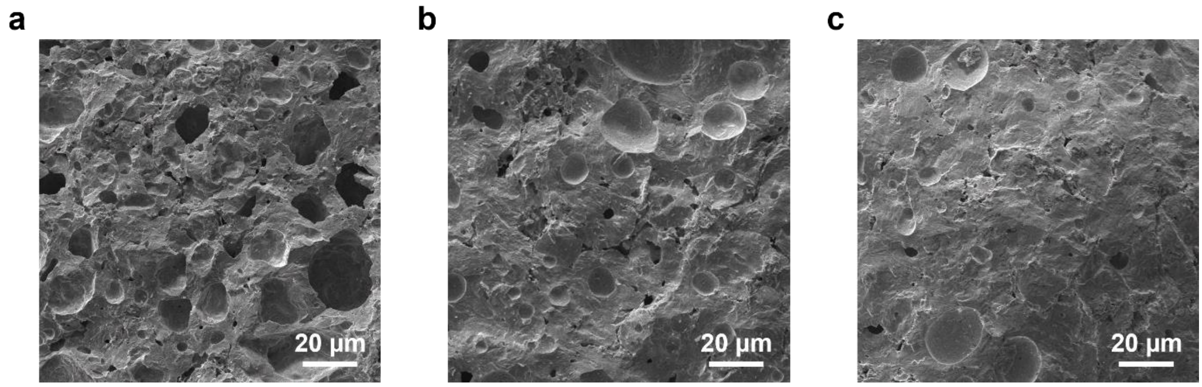

Figure S22. SEM images of 3D-printed a) ABS1, b) ABS3, and c) ABS5 samples.

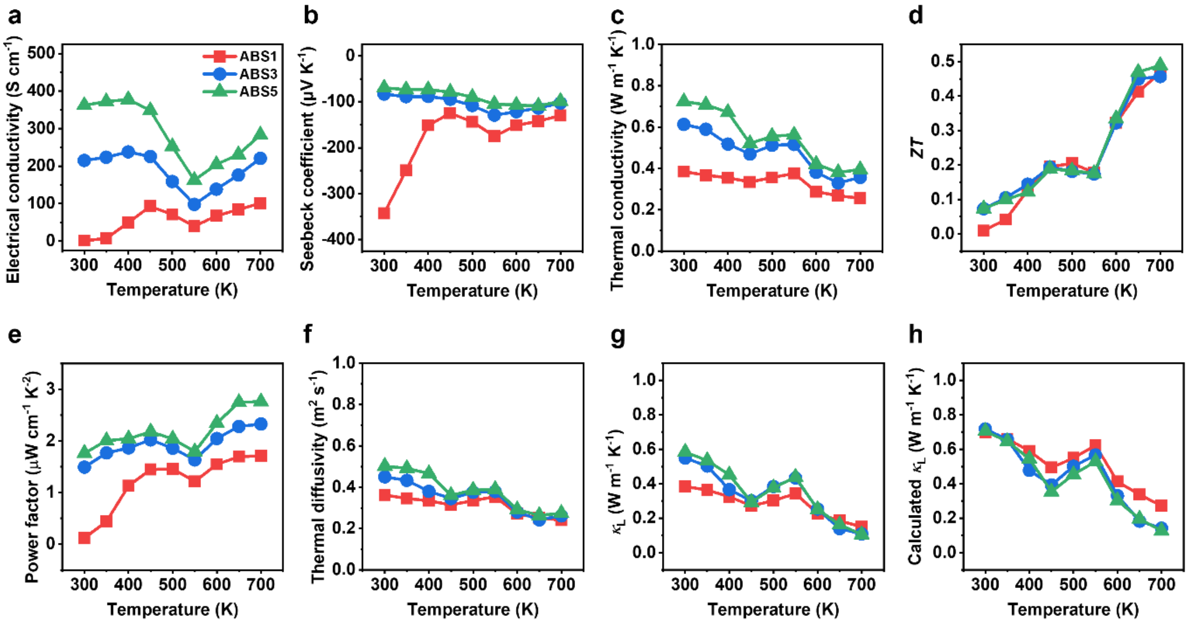

Figure S23. Temperature-dependent a) electrical conductivity, b) Seebeck coefficient, c) thermal conductivity, d) *ZT*, e) power factor, f) thermal diffusivity, g) lattice thermal conductivity and h) calculated lattice thermal conductivity of the 3D-printed ABS1, ABS3 and ABS5 samples.

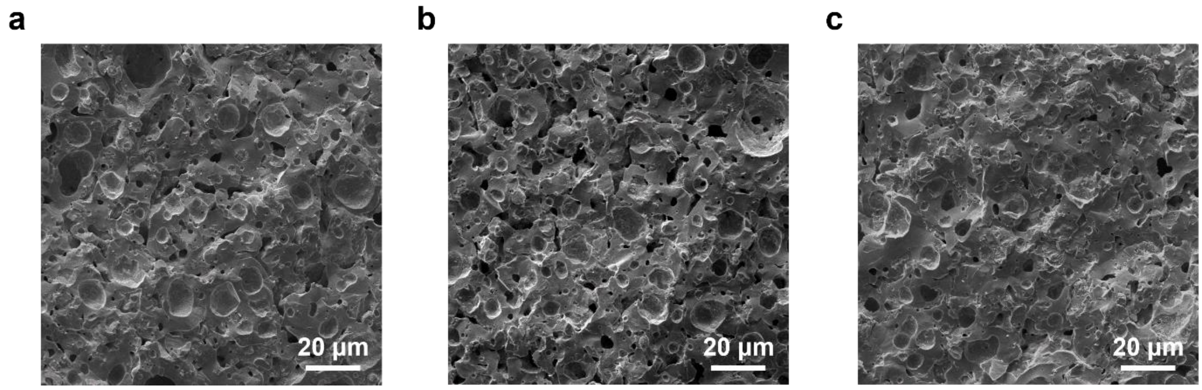

Figure S24. SEM images of 3D-printed a) AST1, b) AST5, and c) AST9 samples.

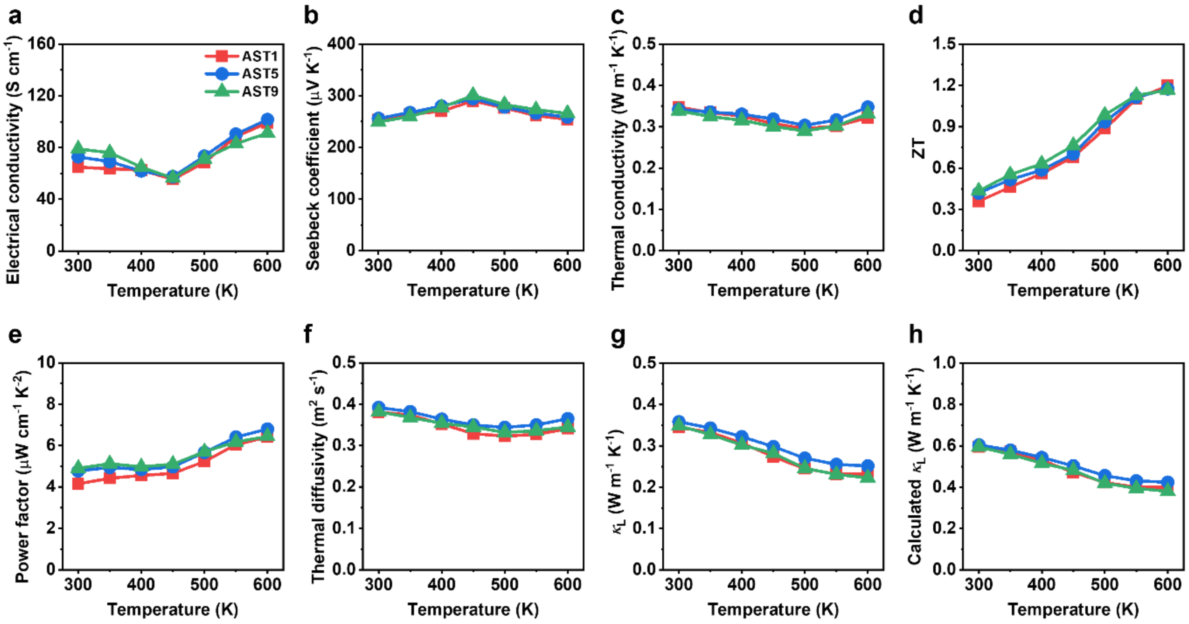

Figure S25. Temperature-dependent a) electrical conductivity, b) Seebeck coefficient, c) thermal conductivity, d) *ZT*, e) power factor, f) thermal diffusivity, g) lattice thermal conductivity and h) calculated lattice thermal conductivity of the 3D-printed AST1, AST5 and AST9 samples.

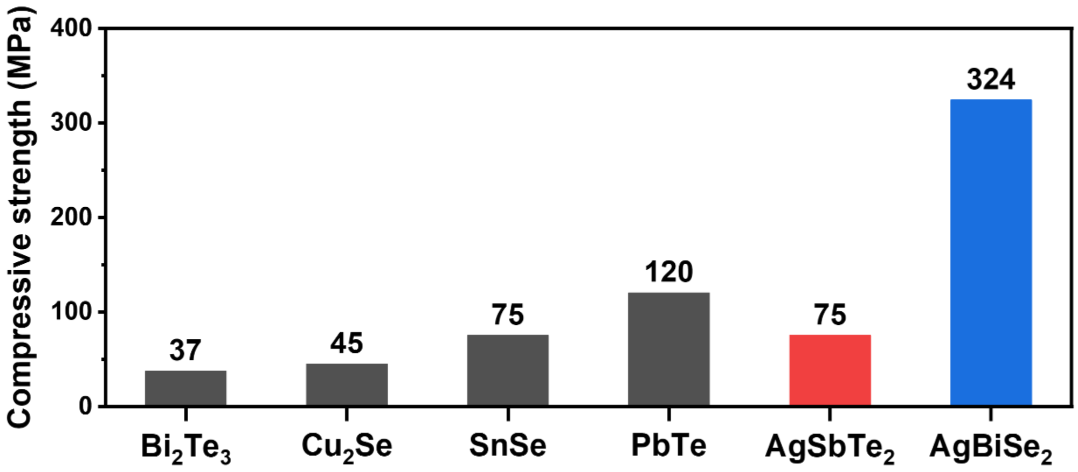

Figure S26. Comparison of the compressive strength values among the 3D-printed ternary Ag chalcogenide TE samples and the reported conventional bulk TE samples.

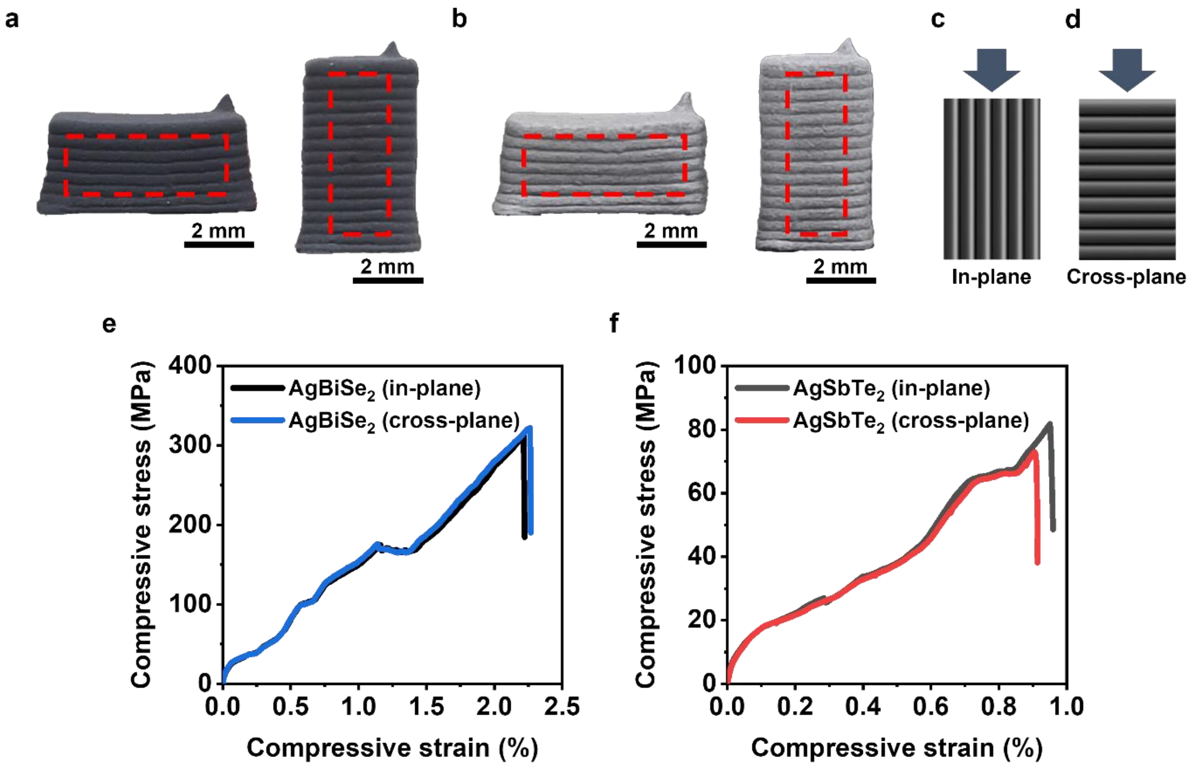

Figure S27. Photographs of 3D-printed cuboid a) $AgBiSe_2$ and b) $AgSbTe_2$ through in-plane and cross-plane printing directions. Compressive stress-strain curve of the 3D-printed c) $AgBiSe_2$ and d) $AgSbTe_2$ samples to compare in-plane and cross-plane printing directions.

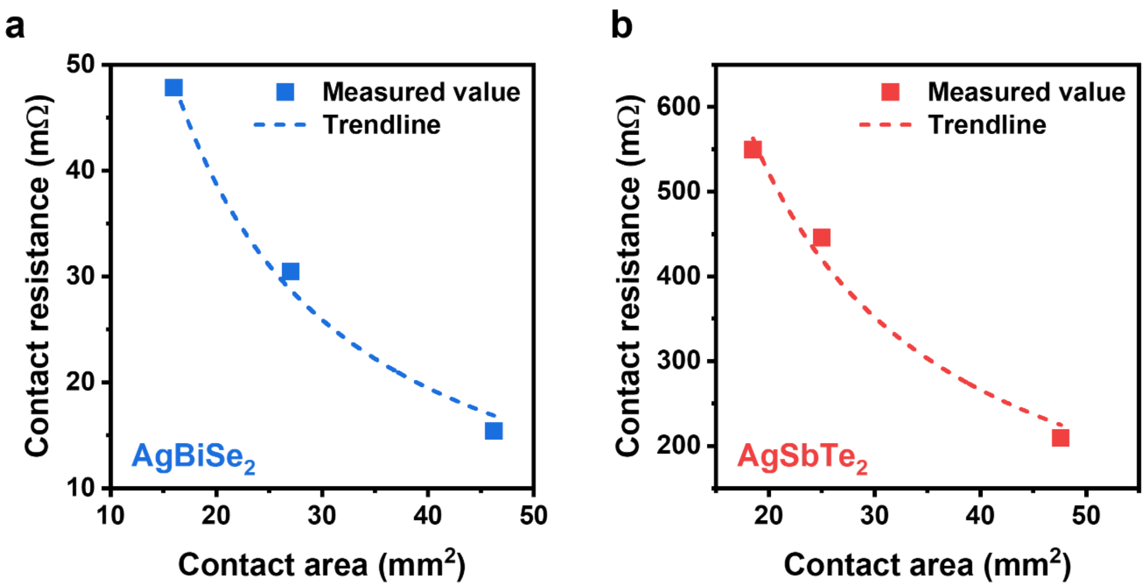

Figure S28. Contact resistance measurements of 3D-printed a) $AgBiSe_2$ and b) $AgSbTe_2$ samples at different contact areas.
